# Dual plasmepsin IX and X inhibitors are refractory to development of resistance

**DOI:** 10.1371/journal.ppat.1014068

**Published:** 2026-09-08

**Authors:** Paola Favuzza, Madeline G. Dans, Wenyin Su, Jennifer K. Thompson, Anthony N. Hodder, Anna Ngo, Jocelyn Sietsma Penington, Danushka S. Marapana, Jerzy M. Dziekan, Anthony T. Papenfuss, Manuel de Lera Ruiz, Rachael Coyle, Marcus C. S. Lee, John A. McCauley, Kym Lowes, David B. Olsen, Brad E. Sleebs, Alan F. Cowman

**Affiliations:** 1 The Walter and Eliza Hall Institute of Medical Research, Parkville, Victoria, Australia; 2 Department of Medical Biology, The University of Melbourne, Melbourne, Victoria, Australia; 3 Merck & Co., Inc., West Point, Pennsylvania, United States of America; 4 Wellcome Sanger Institute, Wellcome Genome Campus, Hinxton, United Kingdom; 5 Biological Chemistry and Drug Discovery, School of Life Sciences, University of Dundee, Dundee, United Kingdom; University of Georgia, UNITED STATES OF AMERICA

## Abstract

Artemisinin-based combination therapies (ACTs) remain the cornerstone of malaria treatment, but emerging resistance threatens their efficacy. The potential for the development of drug resistance against plasmepsin X (PMX)-selective inhibitors and dual plasmepsin IX/X (PMIX/X) inhibitors was investigated in *Plasmodium falciparum*. A series of PMX-selective (WM4, WM76, WM92) and PMIX/X dual inhibitors (WM382, WM09, WM42) were characterised for potency against parasite growth and enzyme inhibition. *In vitro* selection experiments showed that all compounds had a high barrier to resistance, although parasites with reduced sensitivity to PMX‑selective inhibitors could still be selected. Resistance mechanisms involved *pmx* gene amplification and point mutations (D245N, S315P, S359P, I363L) that alter inhibitor binding. Recombinant expression and Michaelis–Menten kinetics demonstrated that these mutations impair drug binding whilst preserving PMX catalytic function. Reverse genetics confirmed that introducing these mutations into the *pmx* gene resulted in decreased potency of the inhibitors. In this study, resistance to the PMIX/X dual inhibitors evaluated here could not be selected, despite prolonged selection pressure. Antimalarial Resistome Barcoding (AReBar) assays confirmed the absence of pre-existing resistance to either inhibitor class. Critically, PMIX/X dual inhibitors maintained efficacy against parasites with decreased sensitivity to PMX-selective compounds. These findings demonstrate that dual PMIX/X inhibitors present a substantially higher barrier to resistance than PMX-selective inhibitors, informing antimalarial drug development strategies and highlighting dual-target inhibition as a promising approach to mitigate resistance risks.

## Author summary

Malaria treatment relies heavily on combination therapies, but drug resistance continues to undermine their effectiveness. We investigated whether *Plasmodium falciparum* could develop resistance to two classes of experimental inhibitors that target parasite aspartic proteases: compounds that selectively block plasmepsin X (PMX) and compounds that simultaneously inhibit plasmepsin IX and X (PMIX/X). PMX-selective inhibitors could, with prolonged laboratory exposure, select parasites with reduced sensitivity driven by amplification of the *pmx* gene and by specific point mutations (S315P, D245N, I363L, S359P) that decrease drug binding yet preserve enzyme function. By contrast, dual PMIX/X inhibitors resisted resistance selection even after extended pressure; no resistant parasites were recovered, and no pre-existing resistance was detected in a diverse panel of resistant lines. Importantly, dual inhibitors retained activity against PMX-altered parasites. Resistance-associated *pmx* changes imposed measurable fitness costs on parasites, reducing their competitiveness without drug pressure. These results indicate that dual-target inhibition markedly raises the barrier to resistance and support prioritising PMIX/X dual inhibitors for antimalarial development to prolong drug utility.

## Introduction

The global fight against malaria has faced significant challenges in recent years, despite notable progress in the early 21st century. From 2000 to 2019, annual malaria deaths declined substantially from 864,000–576,000 [1]. However, the COVID-19 pandemic reversed this positive trajectory, with mortality increasing to 631,000 in 2020 and remaining elevated at 610,000 deaths in 2024–43,000 more than pre-pandemic levels. Similarly, global malaria cases rose from 233 million in 2019 to approximately 282 million in 2024, highlighting the pandemic’s devastating impact on malaria control efforts [1].

Artemisinin-based combination therapies (ACTs) continue to be the cornerstone of malaria treatment, particularly for *Plasmodium falciparum* infections. The scale of ACT deployment is remarkable, with approximately 4.7 billion treatment courses distributed globally between 2010 and 2024, including 260 million in 2024 alone [1]. However, this widespread use has been accompanied by an emerging issue: the development of complex, multifaceted drug resistance mechanisms. Initially identified in Southeast Asia nearly two decades ago, artemisinin partial resistance in *P. falciparum* has now been documented in South America (Guyana), Papua New Guinea, and most critically, has emerged independently in East Africa. Countries including Rwanda, Uganda, South Sudan, Tanzania, Ethiopia, Eritrea, and eastern Democratic Republic of Congo (DRC) have reported established artemisinin partial resistance (reviewed in [2,3]). Artemisinin partial resistance is complex but primarily mediated through mutations in PfKelch13 that affects the protein’s regulatory function in cell stress responses [4,5] that act together with other mutations in proteins involved in the endocytic pathway (reviewed in [6]). The partner drugs of artemisinin in ACTs are also succumbing to resistance, with increased gene copy numbers of *P. falciparum plasmepsin 2* and *3* correlating with piperaquine resistance in Southeast Asia [7–9].

The discovery and development of novel antimalarial drugs have become increasingly important due to the rise of *P. falciparum* resistance to current frontline treatments. Several promising new drug targets are being explored, including *Plasmodium* proteasome inhibitors, which have shown efficacy against both blood and liver stages of the parasite [10]. Other approaches target the parasite’s phosphatidylinositol 4-kinase (PI4K), as exemplified by MMV390048 which entered clinical trials [11], and the enzyme dihydroorotate dehydrogenase (PfDHODH) with compounds like DSM265 showing potential against drug-resistant strains [12]. Another promising candidate is Ganaplacide, that has recently been developed in combination with lumefantrine as GanLum, showing potential as a novel antimalarial treatment [13]. The focus on novel targets is essential because of the ability of *P. falciparum* to evolve new resistance mechanisms. This has spurred increased investment in drug candidates with entirely new mechanisms of action that would potentially circumvent existing resistance pathways [14].

Aspartic proteases in *Plasmodium* species, particularly plasmepsin IX (PMIX) and plasmepsin X (PMX), have emerged as therapeutic targets against malaria [15–19]. These proteases play essential roles in the parasite life cycle, with PMIX being required for merozoite egress through involvement in subtilisin 1 maturation, and PMX functioning as a maturase for SERA5 and SERA6 during schizont rupture [15–17]. Additionally, PMX processes a number of *P. falciparum* proteins that play important roles in merozoite invasion including the essential ligand PfRh5 [20,21]. The indispensable nature of these enzymes for parasite survival, combined with distinct structural features compared to human aspartic proteases, has led to development of new antimalarial compounds, notably with the discovery of WM382, a potent dual inhibitor that simultaneously targets PMIX and PMX [17,18].

Recent structural and biochemical studies have revealed that PMIX and PMX possess distinctive substrate specificity pockets that differ significantly from their human homologs [22]. This structural divergence has been leveraged in drug discovery efforts, leading to the evolution of WM382 to MK-7602, a promising clinical candidate maintaining dual inhibition of PMIX and PMX. The discovery of MK-7602 demonstrates the feasibility of targeting these essential proteases with antimalarials that could lead to new therapeutic options with both curative and transmission-blocking properties. Both WM382 and MK-7602 target the liver (<2 x 100 mg/kg blocks *P. berghei in vivo*), blood (~0.4 nM inhibits asexual stage development *in vitro*) and transmission stages of *P. falciparum* (2–10 nM blocks oocyst formation) and they have a high barrier for the selection of resistance due to their dual targeting of PMIX and PMX [17,19]. The pharmacokinetic profile and tolerability of MK-7602 have been evaluated in two phase 1 studies and support continued clinical development [23]. Additionally, the antimalarial activity of clinical candidate MK-7602 has been tested in a phase 1b Controlled Human Malaria Infection (CHMI) human clinical trial with encouraging results (NCT06294912) (under review).

The analysis of resistance mechanisms for new antimalarial clinical candidates involves a multi-layered approach combining *in vitro* evolution studies, genomic analysis, and clinical surveillance. This comprehensive resistance analysis has become important for early identification of resistance mechanisms to assist in optimizing dosing strategies to minimize resistance development. In *P. falciparum* this approach has successfully identified mutations in PfCARL, PfACT and PfUGT as resistance mechanism(s) for the imidazolopiperazine compound Ganaplacide, and mutations in PfATP4 conferring resistance to several new antimalarial candidates including Cipargamin [24,25].

In this study, the possibility of selecting resistance to PMX selective inhibitors and PMIX/X dual inhibitors by *P. falciparum* was investigated. This included the PMX selective inhibitors WM4, WM76, WM92 that were compared to the dual PMIX/X inhibitors WM382, WM09 and WM42. Our findings demonstrated a high barrier against the selection of resistance in *P. falciparum*; however, it was possible to select decreased sensitivity for the PMX selective inhibitors. The molecular mechanisms of resistance involved amplification of the *pmx* gene and mutations within the protease that alter the ability of the inhibitors to bind and inhibit protease activity were identified. This has contributed to our understanding of potential resistance mechanisms to these promising new antimalarial drug candidates and informs strategies for their optimal development and deployment.

## Results

### Characterization of selective and dual inhibitors targeting *P. falciparum* plasmepsin IX and X

**Fig 1:**
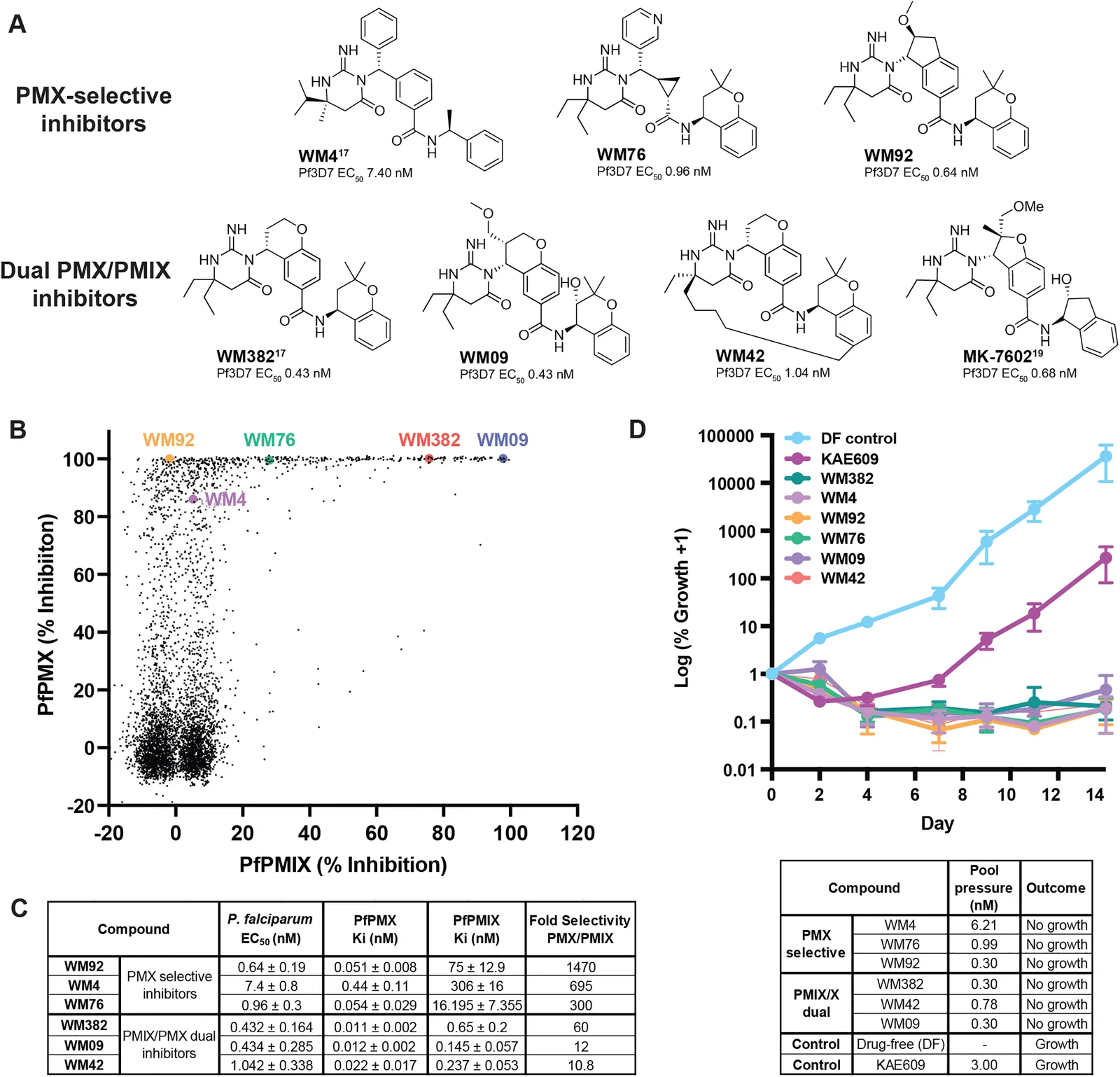
PMX selective and dual PMIX/X inhibitors. **A**. Chemical structures and EC_50_ values of PMX-selective and PMX/IX dual inhibitors. WM4 and WM382 [17] and MK-7602 [19] are previously described. **B.** A library of 4505 aspartic protease inhibitors (10 nM) was screened against recombinant *P. falciparum* PMX (0.2 nM) and PMIX (5 nM) and plotted as percent inhibition of enzyme activity. **C.** EC_50_ for *P. falciparum* and K_i_ for PfPMX and PfPMIX (Mean ± SD, three independent experiments). **D.** PMX-selective and PMIX/X dual inhibitors profiled in AReBar assay at concentrations equivalent to 3x EC_50_, alongside drug-free treatment (DF, light blue) and KAE609 (magenta) as controls. DF control, KAE609, WM4 and WM382 were previously described [19]. Concentrations of compounds used in the experiment (pool pressure) are reported in the lower table.

A high-throughput screen of aspartic protease inhibitors identified iminopyrimidinone compounds that blocked *P. falciparum* growth, including the hit compound WM4 (Fig 1A) [17,18]. This discovery prompted a medicinal chemistry campaign to develop structural analogs of WM4, ultimately leading to the tool compound WM382 and clinical development compound MK-7602, both potent dual inhibitors of PMIX and PMX [19]. The program involved the design and synthesis of approximately 5,000 compounds, including WM382, WM76, WM09, WM92, WM42 and MK-7602 (Fig 1A) [17–19].

All synthesized compounds were systematically evaluated for their inhibitory activity against recombinant *P. falciparum* aspartic proteases PMIX and PMX (Fig 1B) [17]. This extensive screening effort revealed three distinct groups of compounds: those showing little or no inhibitory activity against either PMIX or PMX, PMX-specific inhibitors including WM4, WM76, and WM92, and PMIX/X dual inhibitors WM382 and WM09 (Fig 1B, 1C). Notably, no PMIX-selective compounds were identified during this process (Fig 1B), highlighting the unique properties of these aspartic proteases and challenges in discovering selective inhibitors.

PMX-selective and dual PMIX/X inhibitors demonstrated potent inhibition of *P. falciparum* asexual blood stage growth (Fig 1C), consistent with the essential nature of both PMIX and PMX in the parasitic lifecycle [15–17]. The optimized PMX-selective inhibitors WM92 and WM76 showed significantly increased potency against *P. falciparum* blood stage parasites compared to the hit compound WM4, with EC_50_ values of 0.64 nM and 0.96 nM, respectively (Fig 1C). Similarly, the dual PMIX/X inhibitors displayed comparable potency, with WM09 and WM42 exhibiting EC_50_ values of 0.43 nM and 1.04 nM, respectively. These optimized compounds demonstrated substantially improved potency over the hit compound WM4 (EC_50_ 7.4 nM) (Fig 1C) and were comparable to the tool compound WM382 (0.43 nM) and the clinical candidate MK-7602 [17,19].

To further characterize the compounds, we determined their inhibitory binding constants (K_i_) for PMIX and PMX, providing insight into their potency and selectivity profiles (Fig 1C). The hit compound WM4 selectively inhibits PMX with a K_i_ of 0.44 nM, demonstrating a 695-fold selectivity over PMIX [17,22]. The optimized PMX-selective inhibitors WM76 and WM92 showed even greater potency and selectivity, with PMX K_i_ values of 0.054 nM and 0.051 nM, respectively, and selectivity ratios against PMIX of 300-fold and 1,470-fold (Fig 1C). In contrast, the dual inhibitors WM382, WM09, and WM42 exhibited potent inhibition of both proteases, albeit with varying degrees of selectivity. The lead compound WM382 showed a 60-fold selectivity between PMX and PMIX, while WM09 and WM42 demonstrated K_i_ values for PMX of 0.01 nM and 0.02 nM, with selectivity ratios of 12-fold and 10.8-fold, respectively. These results highlight the development of both highly selective PMX inhibitors and potent dual PMIX/X inhibitors, providing a set of tools to further investigate the function of these enzymes during different stages of parasite replication. Moreover, these compounds provide valuable tools to examine the differential propensity for resistance development in *P. falciparum* between PMX-specific and PMIX/X dual inhibitors.

### PMIX and PMX inhibitors demonstrate no cross-resistance with known mechanisms of drug resistance

To investigate potential cross-resistance between PMX-selective and PMIX/X dual inhibitors and known antimalarial resistance mechanisms, the Antimalarial Resistome Barcoding (AReBar) library of resistant *P. falciparum* parasites was employed (Fig 1D) [26]. This comprehensive approach simultaneously assesses the efficacy of the compounds against a population of 52 *P. falciparum* parasite lines possessing genetically engineered to express resistance mechanisms to a broad range of compounds, accounting for most known resistance mechanisms (S1 Table). EC_50_ values were determined for inhibition of *P. falciparum* Dd2 and 3D7 growth to determine the pool pressure (Fig 1D) to be applied during the experiment.

Over the course of the 14-day AReBar assay, the drug-free control population expanded as expected (Fig 1D). In contrast, parasites exposed separately to 3x EC_50_ of the PMX-selective inhibitors (WM4, WM76, and WM92) and PMIX/X dual inhibitors (WM382, WM09, and WM42) failed to grow, with no viable parasites detected at the end of the selection period. This contrasted with the control compound Cipargamin (also known as KAE609), a PfATP4 inhibitor, which allowed for the expansion of *P. falciparum* lines encoding resistance mechanisms specific to this drug [17,27]. The Cipargamin-resistant parasites were readily detectable by day 14, serving as a positive control for the assay’s ability to identify resistant populations (Fig 1D). The lack of growth in the presence of our PMIX and PMX inhibitors demonstrates that known mechanisms of resistance in *P. falciparum* do not confer any protection against these novel compounds. This outcome, while not entirely unexpected given the unique mechanisms of action of PMIX and PMX inhibitors, provides evidence for their potential to overcome existing in-the-field clinical resistance [17].

### PMX-selective and PMIX/X dual inhibitors have a high barrier to drug resistance development in *P. falciparum*

**Fig 2:**
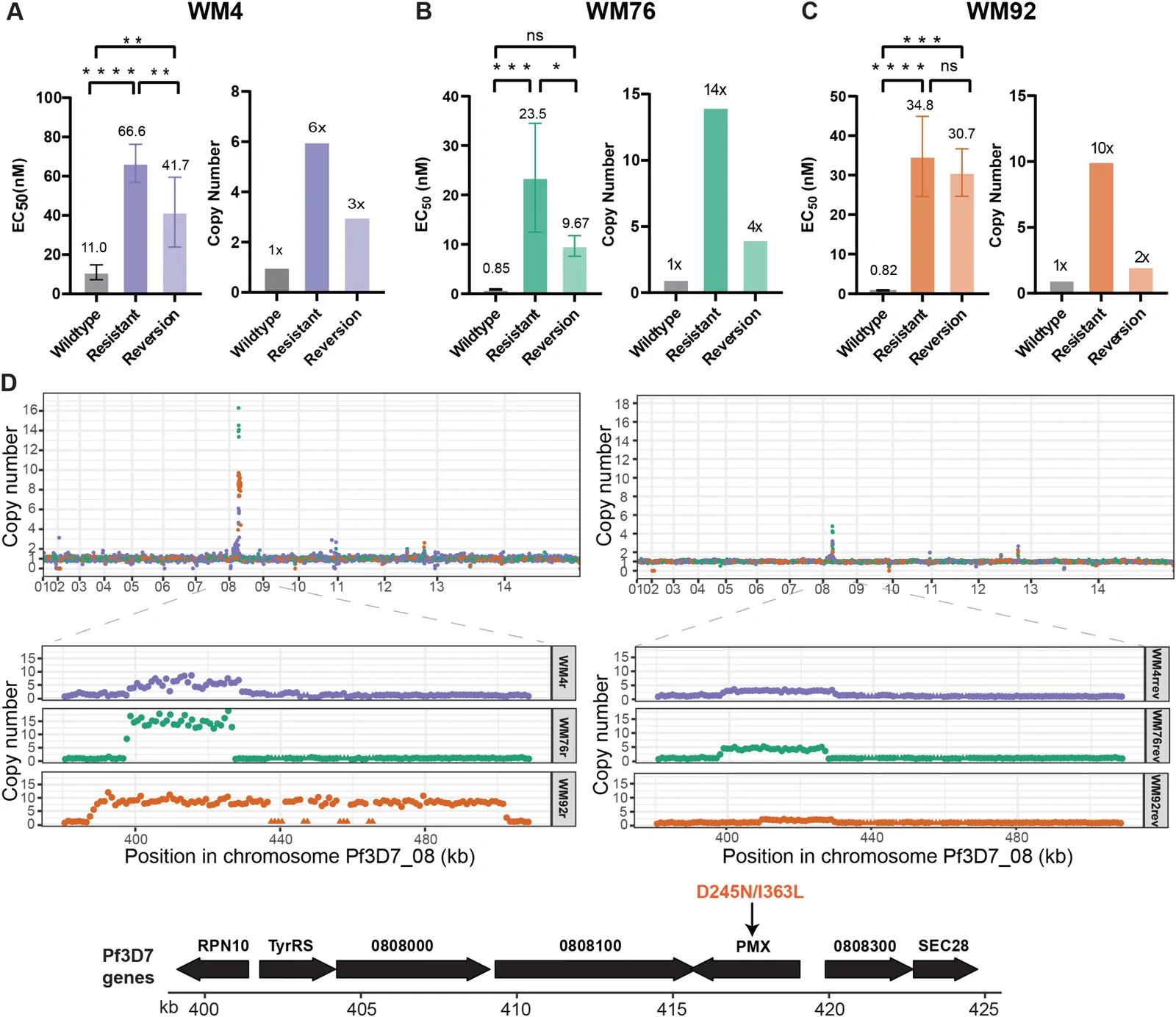
Resistance generated against PMX-selective inhibitors. **A.** Left panel shows the EC_50_ values (nM) derived from growth inhibition assay for wildtype, Pf3D7-WM4-D2-RU (‘Resistant’) and Pf3D7-WM4-D2-RU-REV (‘Reversion’) lines. The right panel shows the *pmx* gene copy number for these parasite lines. **B.** Left panel shows the EC_50_ values (nM) derived from growth inhibition assays for wildtype, Pf3D7-WM76-RU-3 (‘Resistant’) and Pf3D7-WM76-RU-3-REV (‘Reversion’) lines. The right panel shows the *pmx* gene copy number for these parasite lines. **C.** Left panel shows the EC_50_ values (nM) derived from growth inhibition assays for wildtype, Pf3D7-WM92-RU (‘Resistant’) and Pf3D7-WM92-RU-REV (‘Reversion’) lines. The right panel shows the *pmx* gene copy number for these parasite lines. For A, B, and C the error bars represent the SD of >3 independent experiments. Statistical analysis conducted by Ordinary One-way ANOVA with multiple comparisons in GraphPad Prism. * p < 0.05, ** p < 0.01, *** p < 0.001, **** p < 0.0001. **D.** Genome of *P. falciparum* WM4-, WM76- and WM92-resistant (left) and reversion (right) lines showing copy number (top) and detail of amplification of chromosome 8 (bottom). Triangles indicate regions where copy number was not determined. Gene numbers identified as amplified in this region can be found at PlasmoDB: https://plasmodb.org/plasmo/app/. WM4 (purple), WM76 (green), WM92 (orange). The D245N, I363L SNPs identified in the *pmx* gene are highlighted.

PMX-selective and PMIX/X dual inhibitors provided valuable tools to compare the risk of resistance for compounds targeting single versus dual targets in *P. falciparum*. The established method of exposing *P. falciparum* blood stage parasites to drug pressure using a slow, incremental increase in the concentration of each tested compound was employed [28,29]. In a previous study, drug selection with the hit compound WM4 for 6 months using the ramp up (RU) approach, yielded a resistant parasite line (‘Pf3D7-WM4-D2-RU’) with an EC_50_ of 66.6 nM corresponding to a 6-fold shift (Fig 2A) (S2 & S4 Tables) [17]. Using the same approach, parasites with decreased sensitivity to the selective PMX inhibitors WM76 and WM92 were generated (‘Pf3D7-WM76-RU-3’ and ‘Pf3D7-WM92-RU’) (Fig 2B, 2C) over 15 and 3 months, achieving an approximate 28- and 42-fold shift in EC_50_, respectively (WM76-selected EC_50_ 23.5 nM and WM92-selected EC_50_ 34.8 nM) (Fig 2B, 2C). In stark contrast, dual PMIX/X inhibitors WM09 and WM42 demonstrated a resistance profile similar to WM382 rather than the clinical candidate MK-7602, as neither decreased sensitivity nor resistance could be generated even after prolonged selection (Table 1) [17]. Although resistance to MK-7602 has previously been generated, this required an exceptionally high level of gene amplification (20 copies of *pfpmix* and 18 copies of *pfpmx*) and approximately 15 months of continuous selection [19], highlighting the substantial resistance barrier associated with dual PMIX/X inhibition.

**Table 1:** Resistance profile characterization with PMX selective and PMIX/X dual inhibitors.

Compound	Selectivity	Selection time (days)	Maximum EC_50_ fold-pressure	EC_50_ foldshift	Genome variant(s)	Minimum inoculum for resistance
**WM4**	PMX	180	4.5	6	PMX amplification	> 9
**WM76**	PMX	450	8	28	PMX amplification	> 9
**WM92**	PMX	90	20	42	PMX amplificationPMX(D245N,I363L)	8
**WM382**	PMIX/PMX	180/240	2	none	None	> 9
**WM09**	PMIX/PMX	120/47	2	none	None	> 9
**WM42**	PMIX/PMX	90/210	2	none	None	> 9

To elucidate the mechanisms underlying decreased sensitivity to the PMX selective inhibitors, whole genome sequencing was performed on the Pf3D7-WM4-D2-RU, Pf3D7-WM76-RU-3 and Pf3D7-WM92-RU parasite lines. This analysis revealed increased copy number of the genomic region surrounding the *pmx* gene on chromosome 8 in all three lines (Fig 2A–2D, S3 Table). The WM4-selected line showed up to 6 copies of a *pmx*-containing amplicon, consistent with our previous findings [17]. The WM76-selected line exhibited a 14-fold increase in a 15 kb amplicon, with no associated SNPs identified. The WM92-selected line displayed approximately 10 copies of a 28 kb amplicon that included the *pmx* gene. Two SNPs in the *pmx* gene were also identified, resulting in D245N and I363L amino acid changes within the PMX protease (S1 Fig, S3 Table). These findings demonstrated that slow incremental drug pressure could select for decreased sensitivity to PMX-selective compounds through both gene amplification and, in the case of WM92, amino acid changes in the PMX protease. Notably, the inability to select for decreased sensitivity or resistance to dual PMIX/X inhibitors WM382, WM09 and WM42 suggests a significantly higher barrier to resistance for these compounds compared to PMX selective inhibitors.

To assess potential fitness costs associated with *pmx* gene amplification and mutations, the Pf3D7-WM4-D2-RU, Pf3D7-WM76-RU-3 and Pf3D7-WM92-RU lines were cultured in drug-free media for 6 months in ‘reversion’ (REV) experiments. Subsequent drug sensitivity analysis revealed significantly reduced EC_50_ values for WM4 and WM76 against the respective compounds used for selection (Fig 2A, 2B). The Pf3D7-WM4-D2-RU-REV line showed an EC_50_ of 41.7 nM down from 66.6 nM (Fig 2A), while the Pf3D7-WM76-RU-3-REV line exhibited an EC_50_ of 9.67 nM down from 23.5 nM (Fig 2B). Intriguingly, the Pf3D7-WM92-RU-REV line maintained essentially the same EC_50_ (30.7 nM) as the resistant line (34.8 nM) (Fig 2C, S2 Table).

Genome sequencing of each reversion line revealed reductions in *pmx* gene copy number. The Pf3D7-WM4-D2-RU-REV line had a 2-fold reduction (from 6 to 3 *pmx* copies), and the Pf3D7-WM76-RU-3-REV line demonstrated a 3.5-fold decrease (from 14 to 4 copies of the *pmx* gene) (Fig 2A, 2B, 2D). Surprisingly, although the Pf3D7-WM92-RU-REV line did not show a reduced EC_50_ to WM92 compared with the Pf3D7-WM92-RU line (Fig 2C), the reversion parasite line exhibited a 5-fold decrease in *pmx* gene copy number (from 10 to 2 copies of the *pmx* gene) (Fig 2C, 2D, S2 Table). The presence of both mutations in both Pf3D7-WM92-RU and the Pf3D7-WM92-RU-REV lines was confirmed by PCR using *pmx*-specific primers (S1 Fig, left panel) and by Sanger sequencing of the amplified products (S1 Fig, right panels). Whole genome sequencing also demonstrated both D245N and I363L were present in both copies of *pmx* in the Pf3D7-WM92-RU-REV parasites (S3 Table). These findings suggested that the D245N,I363L amino acid change plays a key role in conferring and maintaining decreased sensitivity to WM92, even after a reduction in gene copy number.

During these resistance studies, a ‘long-term selected’ (LTS) parasite line was also generated (‘Pf3D7-WM4-RU-LTS’) with wildtype parasites exposed to increasing amounts of WM4 up to 250 nM for over two years. This resulted in an EC_50_ shift of 156-fold (653 nM compared to 4.20 nM in 3D7) (S2A Fig). Whole genome sequencing of this long-term selected line revealed an approximate 14x copy number increase surrounding the *pmx* gene, with an additional SNV identified as S315P in PMX that was found in ~70% of extra copies of *pmx* (S2B, S2C Fig, S3 Table).

Together, these results indicate that although the selection of resistance to PMX-selective inhibitors was possible, it is difficult to achieve and often unstable. In contrast, resistance to dual PMIX/X inhibitors appears even more challenging to select, reflecting a more robust resistance profile. This highlights potential advantages of multi-target inhibition strategies in antimalarial drug development and underscores the complex interplay between gene amplification and point mutations in conferring drug resistance in *P. falciparum*.

### Determination of the minimum inoculum for resistance

**Fig 3:**
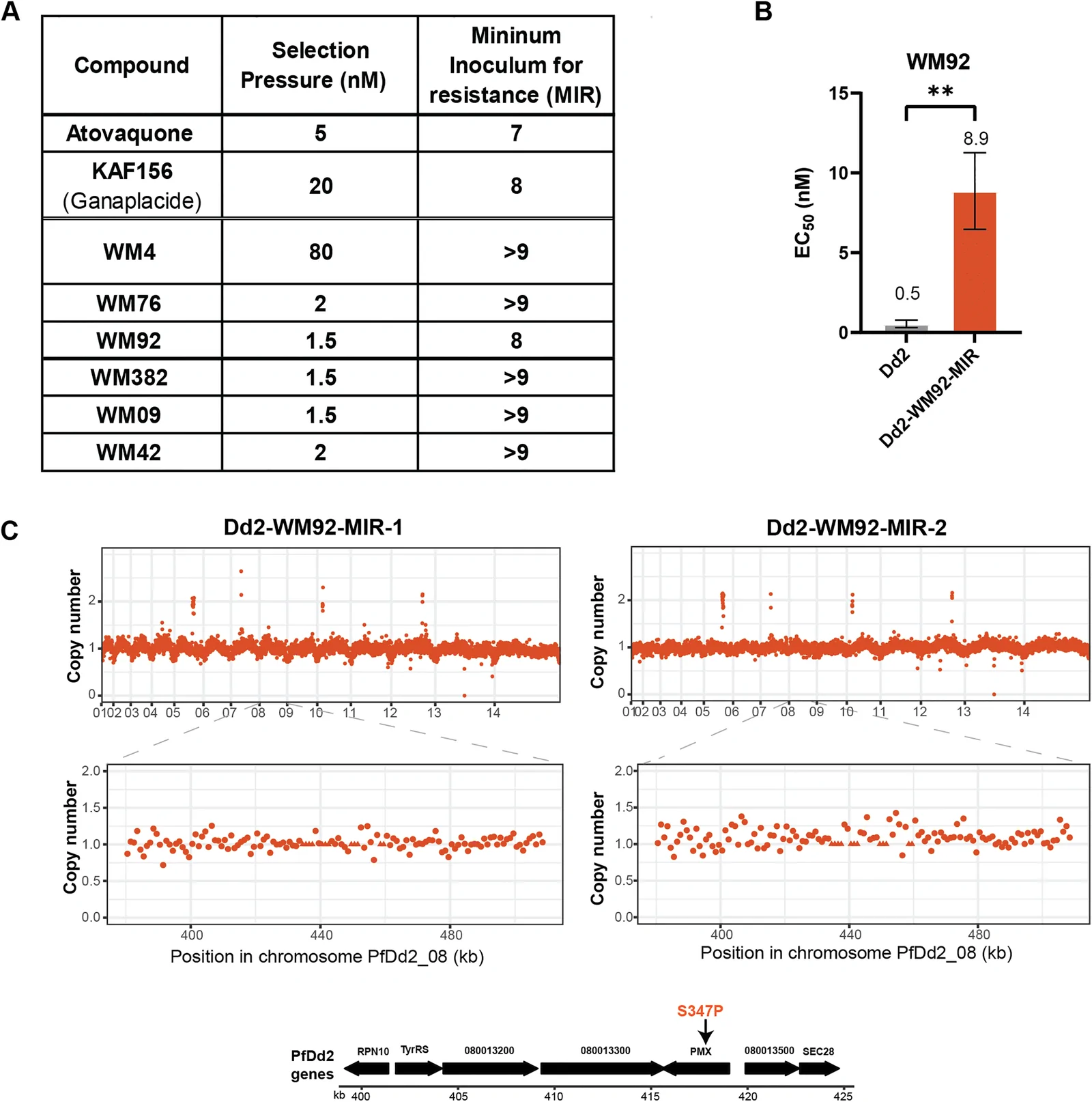
Minimum Inoculum of Resistance (MIR). **A.** Summary of risk of resistance experiments carried out with PMX-selective and PMIX/X dual inhibitors. Emergence of resistance to plasmepsin inhibitors is compared to Atovaquone and Ganaplacide. The initial *inoculum* of *P. falciparum* parasites was varied from 10^7^, 10^8^ or 10^9^ in triplicate, and resistant parasites were selected on a concentration equivalent to the ~ 1x EC_90_ of selected compounds. Recrudescence of parasitaemia was followed by microscopy over 60 days for each experiment respectively. **B.** EC_50_ values (nM) derived from growth curves Dd2 wildtype and PfDd2-WM92-MIR parasite lines for WM92. Error bars represent the SD of >3 independent experiments. Statistical analysis conducted by Ordinary One-way ANOVA with multiple comparisons in GraphPad Prism. ** p < 0.01. **C.** Genomes of PfDd2-WM92-MIR parasite lines derived from two independent cultures initiated at an inoculum of 10⁸ parasites.Whole-genome copy number (top) and detailed region of chromosome 8 (bottom). Gene numbers identified in this region of chromosome 8 can be found at PlasmoDB: https://plasmodb.org/plasmo/app/. The S347P SNP identified in the *pmx* gene is highlighted.

To further evaluate the propensity for the development of resistance, minimum inoculum for resistance (MIR) studies were conducted for WM4, WM76, WM92, and WM382, WM09 and WM42, using the *P. falciparum* Dd2 line. This approach involved exposing a range of *inocula* of parasites to drug pressure for 60 days and determining the smallest parasite *inoculum* from which resistant mutants can be selected [30,31]. The dual PMIX/X inhibitors WM382, WM09 and WM42 demonstrated exceptional barrier to the development of decreased sensitivity. Even at the highest *inoculum* of 10^9^ parasites, no recrudescence was observed over the 60-day selection period, indicating an MIR > 9 (Fig 3A, Table 1). This result underscores the high barrier to resistance for these dual-target inhibitors. Interestingly, the PMX-selective compounds WM4 and WM76 also exhibited a high barrier to resistance in this assay, with no recrudescence observed even with 10^9^ parasites (MIR > 9). This suggests that despite their single-target nature, these compounds maintain a robust resistance profile under the conditions of the MIR assay. In contrast, parasites exposed to the PMX selective inhibitor WM92 at a starting *inoculum* of 10^8^ parasites showed recrudescence (MIR = 8) (‘PfDd2-WM92-MIR’), a result comparable to the control compound Ganaplacide (Fig 3A, Table 1).

The decreased sensitivity of the PfDd2-WM92-MIR parasites was confirmed with standard growth inhibition assays. The selected parasites demonstrated an 18-fold increase in EC_50_ (8.9 nM) compared to the parental Dd2 line (EC_50_ 0.5 nM) (Fig 3B). To elucidate the genetic basis of this decreased sensitivity, whole genome sequencing was performed on two independently WM92-selected dishes (‘PfDd2-WM92-MIR-1/2’) (S2 Table). Unlike the resistance mechanism observed in the incremental selection experiments (performed with 3D7 *P. falciparum* parasites), no copy number variation of the *pmx* gene was detected in the Dd2 WM92-selected line (Fig 3C). However, a small ~2 copy number was detected in chromosomes 10 and 12 of the PfDd2-WM92-MIR parasites (Fig 3C, S3 Table), with 13 unrelated genes amplified in these regions (S5 Table). Amplification events identified on chromosomes 5 and 7 were excluded from further analysis because they were already present in the parental Dd2 strain. Additionally, three SNVs were identified, one in *pmx* that resulted in an S347P amino acid change, which is equivalent to the S359P position in 3D7 parasites (S3 Table). This mutation likely plays a crucial role in conferring decreased sensitivity to WM92. A summary of all resistant lines generated against PMX-selective inhibitors, corresponding genomic variants and phenotypic data can be found in S2- S4 Tables.

### PMX mutants decrease activity of PMX-selective inhibitors

**Fig 4:**
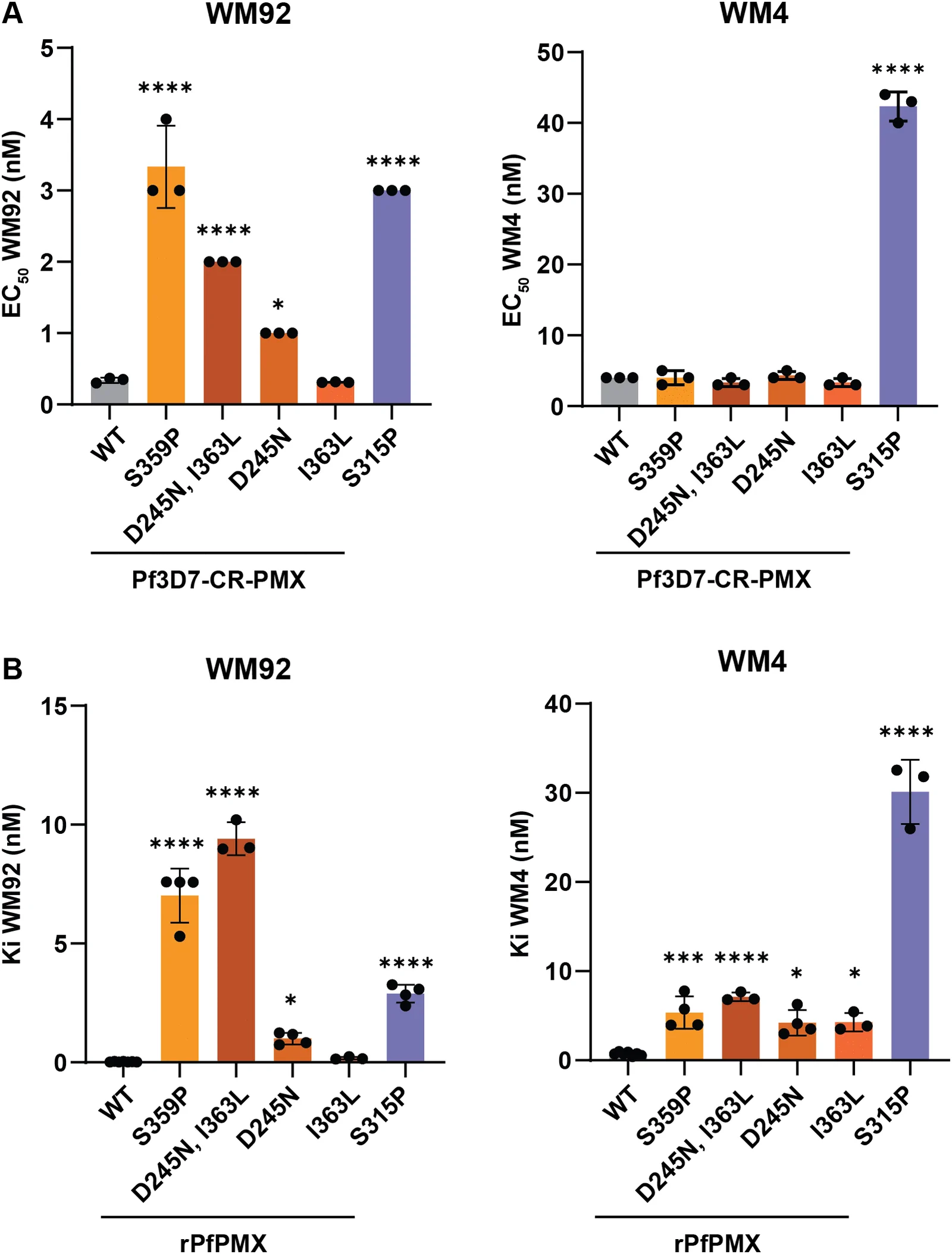
PMX mutations introduced into wildtype *P. falciparum* parasites and recombinant PMX confer resistance to WM92 and WM4. **A.** EC_50_ values for WM92 (left panel) and WM4 (right panel) were determined using growth curves of the CRISPR PMX transfected parasite lines (Pf3D7-CR-PMX) expressing either wildtype (WT) or the amino acid mutations S359P, D245N,I363L, D245N, I363L and S315P. **B.** Recombinant full-length PMX protease (rPfPMX) was expressed and purified in its wildtype form (WT) or with the indicated mutations (S359P, D245N,I363L, D245N, I363L and S315P). The inhibition constant (K_i_) was determined for each protease variant. Error bars represent the SD of >3 independent experiments. Statistical analyses conducted by Ordinary One-way ANOVA with multiple comparisons in GraphPad Prism. * p < 0.05, ** p < 0.01, **** p < 0.0001. ns = not significant.

To investigate the role of the identified SNVs and their contribution to resistance, each was introduced into the *pmx* gene of parental 3D7 parasites (Pf3D7-CR-PMX) (S3A Fig). The presence of the expected nucleotide changes in the *pmx* gene of each genetically modified parasite line was verified by sequencing (S3C Fig). Expression of the resulting mutant proteins were confirmed by western blot using anti-HA antibodies (S3B Fig), demonstrating that SNV insertion did not affect adversely affect PMX protein stability. All transgenic parasite strains were tested in growth inhibition assays to determine EC_50_ values for inhibition by the compounds WM92 and WM4 (Fig 4, S2 and S4 Tables).

Introduction of the S359P mutation, identified in resistant parasites selected through the MIR studies, into the PMX protease caused a significant decrease in sensitivity to the PMX selective inhibitor WM92, with the EC_50_ for parasite growth increasing approximately 10-fold from 0.3 nM to 3.3 nM (Fig 4A). The combination of D245N and I363L mutations, selected through the ramp up approach, in PMX expressed by the transfected parasite line also exhibited reduced sensitivity to WM92, with an EC_50_ of approximately 2 nM. Interestingly, when introduced individually into *P. falciparum*, the D245N mutation caused a modest increase in EC_50_ to approximately 1 nM, while I363L had no significant effect. This suggests an obligate co-evolution between D245N and I363L in conferring decreased sensitivity to WM92. Notably, the S359P, D245N, and I363L mutations when introduced into PMX had no substantial effect on sensitivity of the parasite to the PMX-selective inhibitor WM4, highlighting the compound-specific nature of these resistance mechanisms. In contrast, the S315P mutation, selected under WM4 drug pressure, increased the EC_50_ for both WM92 and WM4, indicating a broader resistance profile due to critical changes in the S2 loop that significantly influence the binding site for these compounds (Fig 4A).

To further analyse the effect of these amino acid changes on PMX protease activity, mutant versions of the enzyme were recombinantly expressed and characterised (rPfPMX) (Fig 4B, S2 and S4 Tables). The S359P mutation in PMX increased the K_i_ for WM92 from 0.1 nM to 7 nM, confirming reduced affinity of binding for the compound (Fig 4B). Similarly, the combination of D245N and I363L mutations increased the K_i_ to 9 nM, while individually these mutations had minimal impact on WM92 binding affinity (Fig 4B). This combination effect at the enzyme level for K_i_ was consistent with the observed increase in EC_50_ in the parasite growth assays.

For the WM4-selected S315P mutation, an increase in K_i_ from 1 nM to 30 nM for the compound WM4 was observed, consistent with the elevated EC_50_ in parasite growth assays (Fig 4A, 4B). The S359P, D245N,I363L, D245N and I363L mutations caused only minor increases in K_i_ for WM4, aligning with their minimal impact on parasite sensitivity to this compound (Fig 4B). These results demonstrate that specific mutations in PMX can confer resistance to PMX-selective inhibitors through reduced binding affinity. The differential effects of these mutations on WM92 and WM4 sensitivity highlight the complexity of drug-target interactions and the potential for compound-specific resistance mechanisms.

### Modelling of compounds in PMX structure

**Fig 5:**
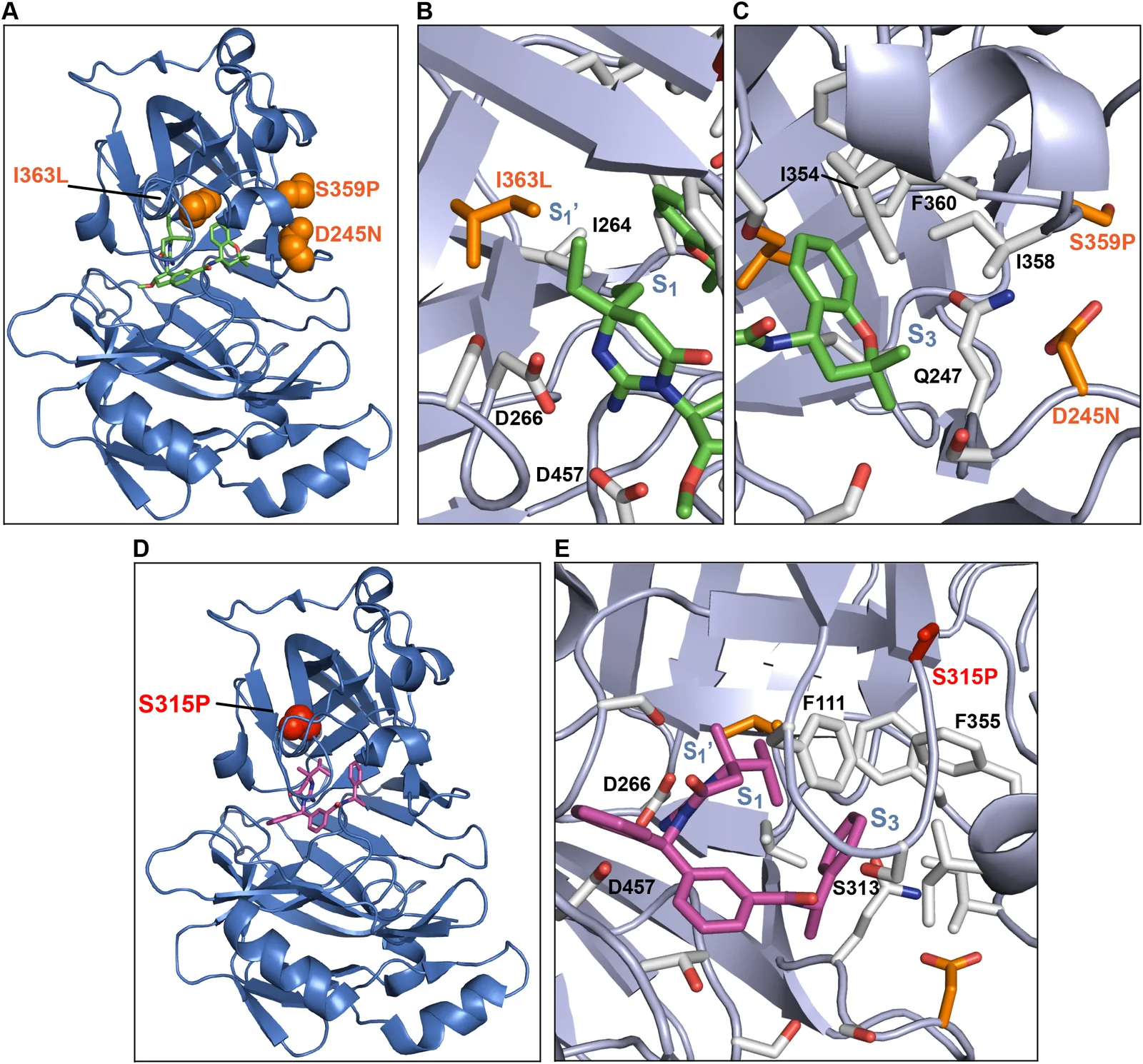
Models showing the location of the PMX resistant mutations found in Pf3D7-WM92-RU and Pf3D7-WM4-D2-RU parasite lines. **A.** The position of the Pf3D7-WM92-RU resistant mutations (orange) surrounding the binding site of WM92 (green) in PMX (blue). **B.** Close-up of the S_1_’ region surrounding the I363L mutation and the P_1_ ethyl group of WM92. **C.** Close-up of the S_3_ region showing the location of the S359P and D245N mutations in relation to the P_3_ chromane group of WM92. WM92 was modelled by building from the structure of WM382 bound to *P. falciparum* plasmepsin X (PDB 7TBC) [22]. **D.** The position of the Pf3D7-WM4-D2-RU mutation S351P (red) located on the flap of plasmepsin X on top of the binding site of WM4. **E.** Close-up of the flap region showing the binding orientation of WM4. WM4 was modelled by superimposing the structure of *P. vivax* PMX in complex with WM4 (PDB 7TBE) with the structure of WM382 bound to *P. falciparum* PMX (PDB 7TBC) [22].

To understand the possible ramifications of the resistant mutations on compound binding, protein models of WM92 and WM4 in complex with *P. falciparum* PMX were created (Fig 5A–5E). The I363L mutation located in the S_1_’ region of PMX is likely to perturb the binding of the P_1_’ ethyl group of WM92 to this pocket (Fig 5B). The D245N and S359P mutations surround the S_3_ pocket, which houses the P_3_ chromane moiety of WM92 (Fig 5C). Although D245 and S359 do not directly interact with the P_3_ chromane moiety, mutations in these amino acids are likely to cause a conformational change in nearby amino acids that interfere with the binding of this moiety to the S_3_ pocket.

Critically, S315 is located on the flap and does not directly interact with WM4 (Fig 5D, 5E), yet a change from Ser to Pro causes a conformational change in the flap structure that perturbs amino acids directly interacting with WM4, reducing its ability to bind to PMX. Notably, S315 represents a non-essential residue in the substrate binding architecture; however, its mutation to P315 causes an increase in Ki of PMX for WM4 indicating that this change decreases the affinity of binding of this compound resulting in an increase in EC_50_ for *P. falciparum* growth assays (Fig 4A, 4B). However, it appears the parasite can tolerate alterations to S315 because it does not participate in the essential interactions required for substrate recognition and proteolytic activity. Conversely, the parasite cannot afford to mutate residues critical for PMX function without compromising parasite replication fitness. Thus, the mutations conferring resistance to WM4 exploit regions of the protease that are functionally malleable for substrate binding, allowing decrease inhibitor binding, while preserving PMX catalytic competence.

### Fitness cost of *pmx* gene amplification and mutations in *P. falciparum*

**Fig 6:**
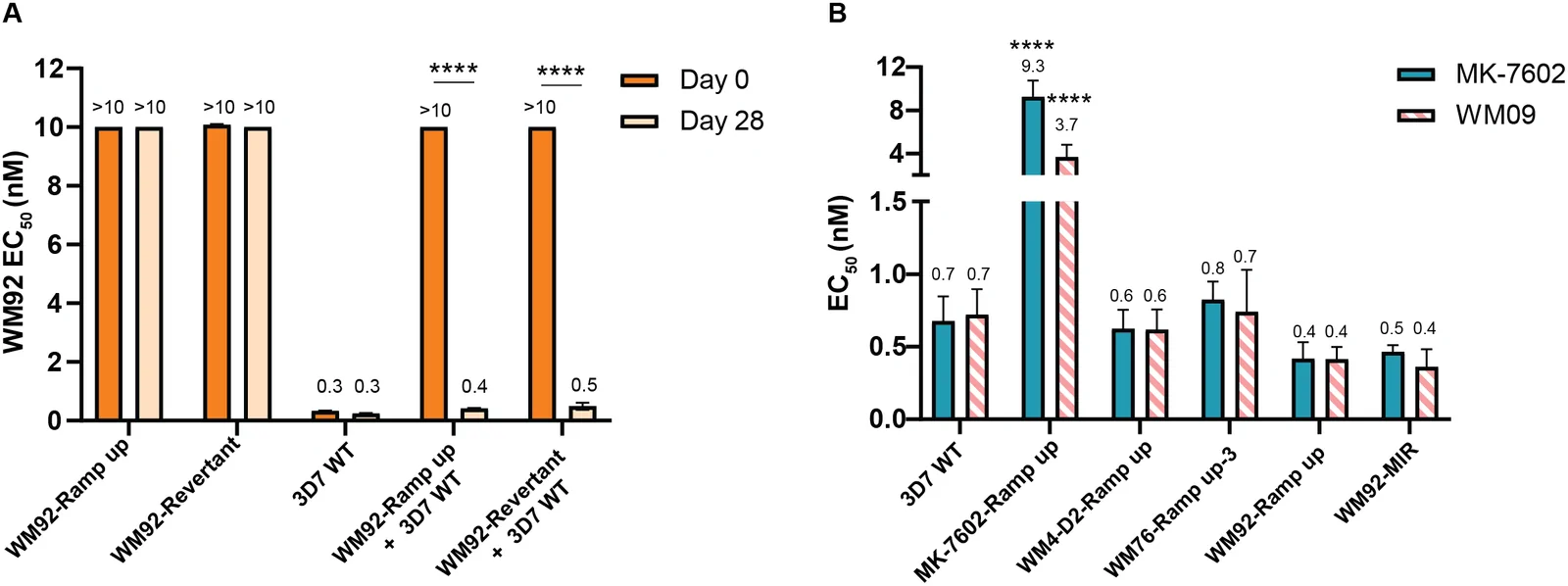
PMX mutant parasites are associated with a reduced parasite fitness and remain sensitive to dual PMIX/X inhibitors. **A.** Pf3D7-WM92-Ramp up (RU) and Pf3D7-WM92-RU-Revertant (REV) parasite lines containing amplifications and mutations in *pmx* were mixed 1:1 with 3D7 wildtype (WT) parasites and the EC_50_ evaluated using LDH growth assays on Day 0 and 28, alongside individual counterpart lines. Error bars indicate the SD of two technical replicates. Statistical analysis was performed in GraphPad Prism via a two-way ANOVA with multiple comparisons. **** indicates p < 0.0001. ns = not significant. **B.** EC_50_ values depicted for dual PMIX/X inhibitors, WM09 and clinical candidate MK-7602, against the PMX mutant lines (Pf3D7-WM4-D2-RU, Pf3D7-WM76-RU-3, Pf3D7-WM92-RU and PfDd2-WM92-MIR) showing sensitivity of the dual inhibitors is maintained. The MK-7602 resistant line, Pf3D7-MK-7602-RU [19], containing a 20x copy number in *pmix* and 18x copy number in *pmx* was included as a control. Error bars indicate the SD of >3 biological replicates. Statistical analysis was performed in GraphPad Prism via a one-way ANOVA with comparison to 3D7 WT. **** indicates p < 0.0001. No asterisks indicate no significance.

To assess the potential fitness costs associated with *pmx* gene amplification and mutations in the aspartic protease, growth competition assays were performed. These assays involved mixing equal number of wildtype and resistant parasites, culturing them over 28 days, and measuring EC_50_ values at the start and at the end of the experiment (Fig 6A). When cultured individually, the Pf3D7-WM92-RU (10x *pmx* amplification and D245N,I363L mutations), Pf3D7-WM92-RU-REV (2x *pmx* amplification and D245N/I363L mutations), and parental 3D7 strains maintained stable EC_50_ values throughout the 28-day period, as expected. However, in direct competition, both Pf3D7-WM92-RU and Pf3D7-WM92-RU-REV parasite lines showed significant fitness disadvantages compared to the parental 3D7. When Pf3D7-WM92-RU parasites were mixed with an equal number of 3D7 parasites, the initial EC_50_ of >10 nM decreased substantially to 0.4 nM by day 28, approaching that of 3D7 alone (Fig 6A). Similar results were observed for the Pf3D7-WM92-RU-REV. Notably, the similar outcomes for both the Pf3D7-WM92-RU and Pf3D7-WM92-RU-REV lines in the competition assays suggest that the fitness cost of the mutations in PMX(I363L,D245N) was significant compared to that of the gene amplification, as the revertant strain maintains the mutations but has reduced copy number.

The same fitness cost experiment was repeated with the engineered Pf3D7-CR-PMX parasite lines containing the introduced mutations: PMX(D245N), PMX(I363L) and PMX(D245N, I363L), using PMX(WT) as the benchmark for normal parasite growth (S4 Fig). This direct growth competition experiment showed that PMX(D245N) and PMX(D245N, I363L) had moderate levels of fitness cost with a 2.3-fold and 2.7-fold reduction in EC_50_, respectively, after 28 days from being mixed 1:1 with PMX(WT) parasites (S4 Fig). In contrast, PMX(I363L) alone showed no fitness cost with equal EC_50_ values observed on day 0 and 28 (EC_50_ = 0.2 nM) (S4 Fig). However, as PMX(I363L) exhibits an EC_50_ comparable to wildtype it may still be outcompeted, though any difference would likely be below the level of detection. These findings demonstrated that the genetic changes in *pmx*, including both gene amplification and mutations, impose a fitness cost on the parasite, with the greatest impact observed with gene amplification of *pmx.*

The relationship between the resistance mechanisms selected by PMX-selective compounds (WM4, WM76, and WM92) and dual PMIX and X inhibitors WM09 and MK-7602 were further investigated. Accordingly, growth inhibition assays were conducted to evaluate potential cross-resistance between parasite lines containing variants in PMX and dual PMIX and X inhibitors. Importantly this revealed that the Pf3D7-WM4-D2-RU (6x *pmx*), Pf3D7-WM76-RU-3 (14x *pmx*), and Pf3D7-WM92-RU (10x *pmx*, D245N, I363L) lines all remained sensitive to both MK-7602 and WM09 dual inhibitors (Fig 6B, S4 Table). In contrast, a previously described MK-7602 selected parasite line (‘Pf3D7-MK-7602-RU’) with amplified copies of both *pmx* and *pmix* [19] showed shifts in EC_50_ values compared to a 3D7 line of 15-fold and 4-fold against MK-7602 and WM09, respectively.

A particularly noteworthy observation was the differential response of the Pf3D7-WM4-D2-RU parasite line to PMX-selective versus PMIX/X dual inhibitors. This strain, characterized by increased PfPMX expression, demonstrated a 6-fold increase in EC_50_ for WM4 (66.6 nM compared to 11 nM in 3D7 WT), a 2.7-fold increase for WM92 (2.25 nM compared to 0.82 nM in 3D7 WT) and 4.1-fold increase for WM76 (3.5 nM compared to 0.845 nM in 3D7 WT) (S4 Table). However, the PMIX/X dual inhibitors WM382, WM09 and MK-7602 maintained their potency against this line, with EC_50_ values (~0.6 nM) nearly identical to those observed in the parental 3D7 strain (0.5 nM, 0.7 nM and 0.7 nM, respectively). The fact that dual inhibitors retained their efficacy against the Pf3D7-WM4-D2-RU line underscores the advantage of targeting both PMIX and PMX simultaneously, as it mitigates the impact of PMX overexpression as a resistance mechanism.

This comparison highlights the distinct resistance profiles of PMX-selective and dual PMIX/X inhibitors and underscore two critical points. Firstly, the genetic changes conferring resistance to PMX-selective inhibitors come at a significant fitness cost to the parasite, potentially limiting the spread of such resistant strains in the absence of drug pressure. Secondly, dual PMIX and X inhibitors maintain their efficacy against parasites with PMX-specific resistance mechanisms, demonstrating both the importance of PMIX in their mode of action and their high barrier to resistance. This robustness persists even in the face of substantial genetic alterations to PMX, reinforcing the potential of dual-target inhibition as a strategy for antimalarial drug development.

## Discussion

The emergence and spread of antimalarial drug resistance poses a major threat to global malaria control efforts. In this context, our data provide important insights into the resistance profiles of PMX-selective versus dual PMIX/X inhibitors and highlight the potential advantages of multi-target inhibition [17,19]. Both inhibitor classes exhibit a relatively high barrier to resistance; however, the dual PMIX/X inhibitors display a clearly superior profile. Even after prolonged *in vitro* drug pressure, we were unable to select parasites with stable resistance to the dual inhibitors, whereas resistance to PMX-selective compounds could be generated, although only with considerable difficulty. Previous work on PMX-selective series has shown that resistance to the compound UCB7362 can be obtained at a minimum *inoculum* (MIR) of 9.6 × 10^6^, and that a related analogue (compound 3) can select resistance at an *inoculum* of 5.3 × 10^5^, albeit with only a modest EC_50_ shift between parental and mutant lines [32]. These findings suggest that the ease of resistance selection is influenced not only by the essentiality of PMX, but also by features of the compound scaffold.

When placed in the broader context of antimalarial development, the resistance profile of dual PMIX/X inhibitors appears particularly robust. For several clinical-stage candidates, including Ganaplacide, Cipargamin, and DSM265, resistant parasites have been selected within 1–4 months of drug pressure, either through single-step selection at high drug concentrations or following prolonged exposure to incrementally increasing sub-lethal concentrations [12,24,33]. The acylguanidine MMV688533 shows a lower propensity for resistance, yet mutations in PfACG1 and PfEHD still confer measurable reductions in potency [34]. By contrast, under comparable or more prolonged *in vitro* selection conditions, we were unable to derive stable resistant lines to dual PMIX/X inhibitors, indicating a substantially higher barrier to resistance. Consistent with this observation, we assessed the resistance liability of these compounds using the *in vitro* candidate selection risk assessment framework, which has been proposed as a standardised approach for prioritising next-generation antimalarials [35]. Based on the resistance-selection data generated in this study, all dual PMIX/X inhibitors and the majority of PMX-selective inhibitors would be categorised as low-risk compounds. WM92 represented the sole exception and would be classified as a medium-risk compound. These findings compare favourably with several clinically deployed antimalarials and highlight the high barrier to resistance associated with PMIX/X inhibition. Collectively, our results further strengthen the rationale for developing dual PMIX/X inhibitors as durable components of future combination regimens aimed at delaying or preventing the emergence of clinical resistance.

This robustness is particularly noteworthy when compared to the resistance profiles of current front-line antimalarials. Artemisinin partial resistance, mediated primarily through mutations in the K13 propeller domain, has emerged and spread relatively quickly in Southeast Asia [4]. Similarly, resistance to partner drugs such as piperaquine has developed through amplification of *plasmepsin 2* and *3* genes and mutations in PfCRT [7,36]. Importantly, the Chloroquine-resistant PH1263-C parasite line, which carries multiple mutations including a *pmII* amplification, remained fully susceptible to all tested PMX- and PMIX/X dual-inhibitors (S4 Table). The high barrier to resistance observed with dual PMIX/X inhibitors is more comparable to that seen with some HIV antiretrovirals, such as second-generation integrase strand transfer inhibitors like dolutegravir [37].

A critical conclusion from this study is that PMX-selective compounds should not be prioritised as antimalarial agents. Although they are potent and well-behaved chemically, their clinical use would focus selection pressure solely on PMX and risk undermining the major advantage of dual PMIX/X inhibitors and an exceptionally high barrier to resistance. We show that PMX-selective compounds can drive both *pmx* gene amplification and the emergence of point mutations in the PMX protease. These adaptations enable parasites to tolerate otherwise inhibitory PMX-targeted pressure. Importantly, however, the resistance phenotypes are not uniform across the PMX-selective series: individual mutations confer resistance to the selecting compound but only partial, or no resistance to other PMX-selective inhibitors. This pattern was consistent with subtle, chemotype-specific differences in how individual inhibitors engage the PMX active site and how particular amino acid substitutions perturb those interactions. Nevertheless, from a population perspective, widespread use of any PMX-selective agent would be expected to seed a diversity of *pmx* alleles, through amplification and mutation, that could collectively lower the evolutionary barrier to resistance across the class and partially diminish the robustness of dual PMIX/X inhibitors by weakening one arm of their dual-target mechanism. Importantly, interrogation of 5,970 *P. falciparum* genomes from the MalariaGEN *Plasmodium falciparum* Community Project, encompassing isolates from 28 malaria-endemic countries, revealed that none of the PMX resistance-associated variants identified in this study (S315P, D245N, I363L, and S359P) were present in the dataset. This finding indicates that these mutations are not currently circulating at detectable frequencies in natural parasite populations and supports the notion that PMX resistance requires specific selective pressure to emerge. Notably, even in the presence of PMX variants and *pmx* amplification, dual PMIX/X inhibitors retain high activity, because they also target the essential protease PMIX and multiple essential PMIX-dependent processes. This underscores the intrinsic resilience of dual-target inhibition and supports a deployment strategy that prioritises dual PMIX/X inhibitors while avoiding clinical use of PMX-selective agents.

The mechanisms of resistance to PMX-selective inhibitors involved both gene amplification and point mutations in the PMX protease. This finding aligns with previous observations of gene amplification as a resistance mechanism in *Plasmodium*, such as the amplification of *pfmdr1* in mefloquine resistance [29,38] and plasmepsin 2 and 3 in piperaquine resistance [7]. The identification of specific point mutations (S315P, D245N, I363L, S359P) provides valuable insights into the molecular basis of drug-target interactions and resistance development. Interestingly, our results revealed compound-specific resistance profiles even among structurally related PMX-selective inhibitors. While resistance selection and forward genetic analyses were performed on only a subset of compounds in this study, expanding these investigations to a broader panel of PMX-selective analogues would provide greater insight into the relationship between compound structure and resistance propensity. Future approaches, including deep mutational scanning of PMX, may further define the structural determinants that influence compound susceptibility and resistance evolution. Nonetheless, this highlights the complexity of drug-target interactions and emphasizes the need for careful consideration of resistance profiles during drug development.

The structural modelling of PMX in complex with the inhibitors provides insights into the molecular mechanisms of resistance and offers guidance for future drug design strategies [22]. The identified mutations appear to affect inhibitor binding through various mechanisms, including steric hindrance, conformational changes, and alterations in pocket geometry. These findings align with previous studies on protease inhibitor resistance in other systems, such as HIV protease inhibitors [39].

The fitness cost associated with PMX amplification and mutations is a crucial finding of the study. Competition assays demonstrated that resistant parasites were significantly outcompeted by wild-type parasites in the absence of drug pressure over 28 days. This fitness cost could potentially limit the spread of resistant parasites in natural populations [40]. The cross-resistance studies, including the Antimalarial Resistome Barcoding (AReBar) assays, revealed no pre-existing resistance to either PMX-selective or PMIX/X dual inhibitors among known resistant *P. falciparum* strains. This lack of cross-resistance with existing antimalarials shows that these compounds could be effective against current drug-resistant parasite populations [9].

The inability to design PMIX-selective inhibitors during the medicinal chemistry program was noteworthy and likely reflects fundamental differences in the structural architecture of the S’ binding sites between PMIX and PMX. The large loop structure in PfPMX, and its associated conformational mobility, likely creates a more permissive S’ pocket that tolerates substantial structural variation. Conversely, dual PMIX/X inhibitors achieve their broad efficacy by concentrating their interactions within the S pockets - regions that are typically more critical for substrate binding and thus more structurally conserved across both proteases.

In conclusion, our findings strongly support the development of dual PMIX/X inhibitors as promising antimalarial candidates with a high barrier to resistance. The superior resistance profile of these dual inhibitors, combined with their efficacy against existing drug-resistant strains, positions them as valuable tools in the ongoing fight against malaria. As we move forward, it will be essential to focus on dual inhibitor development, continue monitoring for potential resistance mechanisms, and explore combination therapies that could further enhance the longevity and efficacy of these promising antimalarial compounds [9].

## Materials and methods

### Ethics Statement

Use of human blood and serum was approved by the Walter and Eliza Hall Institute of Medical Research Human Research Ethics Committee (approval no. 23–05VIC-09). Blood components were supplied by the Australian Red Cross Blood Service as anonymised, de‑identified material; written informed consent for donation and potential research use was obtained by the Blood Service at the time of donation, and no donor-identifiable information was available to the investigators.

### *P. falciparum* culture

*P. falciparum* asexual blood stage parasite cultures and the parasite lines derived from these by genetic manipulation were grown in *in vitro* culture [41]. All parasites were supplied with O^+^ erythrocyte (Australian Red Cross Bloodbank, South Melbourne, Australia) at 4% hematocrit in Roswell Park Memorial Institute (RPMI) 1640 medium supplemented with 26 mM 4-(2-hydroxyethyl)piperazine-1-ethanesulfonic acid (HEPES), 50 μg/ml hypoxanthine, 20 μg/ml gentamicin, 2.9% NaHCO_3_, and 0.5% Albumax II (Gibco) + 5% heat-inactivated human serum or 1% Albumax II (Gibco). Cultures were incubated at 37 °C in a gaseous mix of 94% N_2_, 1% O_2_ and 5% CO_2_. Parasites were sub-cultured with new culture media every 2 days to maintain 4% haematocrit and keep the parasitemia below 5%.

### Construction of *P. falciparum* lines expressing HA-tagged proteins

Transgenic parasite lines were made using the CRISPR-cas9 system as previously described [17]. This involved generation of a guide plasmid and a homology directed repair (HDR) plasmid that replaces the endogenous target gene with a version carrying the desired SNPs and three haemagglutinin tags for validation of gene targeting. The guide plasmid was created by InFusion cloning of the guide oligos MD1/MD2 into *Btg*ZI restriction enzyme digested pUF1-Cas9G vector. These oligos induce a Cas9 dependent double-stranded break at position 496 of the PfPMX protein coding sequence. A modified p1.2 based plasmid encoding WR99210 resistance was used for HDR plasmid creation. Firstly, a 532 bp 3’ homology arm of PfPMX was PCR amplified from *P. falciparum* 3D7 strain genomic DNA using the oligos MD3/MD4 and inserted into the vector using the restriction enzymes *EcoRI* and *Pst*I. This intermediate vector was subsequently modified by inserting the synthetic gene fragment consisting of a combined 5′ homology arm and codon-optimised sequence, containing the desired gene mutations and epitope tag (Genscript), using the restriction enzymes *Not*I and *Xho*I. 50 mg of linearized HDR plasmid and 100 mg of circular guide plasmid were transfected simultaneously into *P. falciparum* parasites that had been previously synchronised using 5% sorbitol and purified using a Percoll gradient to purify schizont stages. This was performed by layering cultured, synchronized *P. falciparum*-infected red blood cells over a pre-formed 35%/65% Percoll step gradient, followed by centrifugation (1,500×g, 15 min, no brake) to separate mature, lower-density schizonts at the interface. Parasites with an integrated drug resistance cassette were selected and maintained on 2.5 nM WR99210. Integration was confirmed via PCR, Sanger sequencing (AGRF) and anti-HA-HRP (1:2000, ThermoFisher) western blotting.

### *P.* *falciparum* growth inhibition assays and EC_50_ determination

The *P. falciparum* growth inhibition assay was conducted with minor modifications [42]. An inoculum of synchronized ring-stage parasitized red blood cells (pRBC) at 0.7% parasitemia and 2% haematocrit in complete media was used for the assay. Assay plates (Greiner #781098, 384 well, white, tissue culture treated) in 16-point dilution series of the compounds were prepared in assay plates using an Echo555 (Labcyte). Appropriate volumes of 10 mM compound stocks were transferred into the assay plates in duplicates, such that the starting concentration was 11.25 µM, with a 1:3-fold dilution series. All wells were backfilled with DMSO to 45 nL such that this remained constant (0.1% DMSO). The growth control was 0.1% DMSO and the negative growth control was 2.5 µM chloroquine. A parasite inoculum (40 μL) was dispensed into plates containing compounds. Following incubation for 72 hours, plates were sealed with parafilm and frozen at -80°C overnight. Plates were thawed at room temperature for at least 4 hours prior to lactase dehydrogenase (LDH) activity being measured. 45 μL of fresh LDH reaction mix (174 mM sodium l-lactate, 214 µM 3-acetyl pyridine adenine dinucleotide (APAD), 270 µM Nitro Blue tetrazolium chloride (NBT), 4.35 U ml^-1^ diaphorase, 0.7% Tween 20, 100 mM Tris-HCl pH 7.5) was dispensed into each well using a Multidrop Combi dispenser at high speed to allow good mixing. Absorbance at 650 nm was measured in an EnVision (PerkinElmer) plate reader after 20 min of incubation at room temperature. Data were normalized to percent viability using positive and negative controls. EC_50_ values (relative inflection of dose response curve) were calculated by Dotmatics 5.3 and Spotfire 7.11.1 software using a nonlinear regression four-parameter fit analysis. The equation used is sigmoidal dose response (variable slope), Y = Bottom + (Top – Bottom) / (1 + (EC_50_ / X)^HillSlope^).

For all other experiments, the determination of EC_50_ values was obtained using ring stage parasites at 0.5% parasitemia which were dispensed in a 50 µL culture at 2% haematocrit in 96 well round bottom microtiter plates (Falcon) with doubling dilutions of each compound. After 72 hours incubation at 37˚C each well was fixed at room temperature for 30 min with 50 µL of 0.25% glutaraldehyde (Pro-SciTech) diluted in PBS. Following centrifugation at 1200 rpm for 1 min, supernatants were discarded, and parasites were stained with 50 µL of 2.5-5X SYBR Green (Invitrogen) diluted in PBS. 50,000 cells were counted by flow cytometry using a Attune Nxt Flow Cytometer (ThermoFisher) or Cell Lab Quanta SC - MPL Flow Cytometer (Beckman Coulter). Growth was expressed as a percentage of the parasitemia obtained using a vehicle control. All samples were tested in triplicate. EC_50_ values were determined using GraphPad Prism.

### Expression and purification of PfPMIX and PfPMX mutant proteases

The apo form of PfPMX wild type (T27 to N573) and the corresponding single mutants PfPMX S315P, PfPMX S359P, PfPMX D245N, PfPMX I363L and the double mutant PfPMX D245N/I363L were expressed in HEK expi 293 cells, using the pTriex2 vector with the inserted gene sequences codon optimised for expression in mammalian cells (Bioneer) [17]. Sequence corresponding to the TEV-FLAG peptide was inserted 5’ to the stop codon to assist with the purification of the expression product and removal of the FLAG peptide tag. Expression of the various PfPMX proteins was conducted as per the manufacturer’s protocols. Culture supernatants were harvested at day 4 of culture (viable cell density of 72%). PfPMX proteins were extracted from the supernatant using M2 antiflag affinity resin (Sigma). Bound protein was eluted using 3 column volumes of 0.1 mg/ml flag peptide. Fractions containing the desired expression product were pooled then concentrated and further purified using a Superdex 200 10/30 size exclusion column (Cytiva) run in the presence of 20mM Tris pH7.2/150mM NaCl. The eluted fractions were analysed using SDS-PAGE and those containing the highly purified PfPMX proteins were pooled for further studies. The produced versions of PfPMX had similar autolytic products to those observed by previously [17,43].

PfPMIX was expressed as previously described [22]. Briefly, to express recombinant PfPMIX the gene sequence (37KDC.NNL627) was re-codoned for insect cell expression and synthesised by GenScript and subcloned into the p1TF vector that enabled expression in insect cells. PfPMIX was expressed in CHO cells as described [44]. PfPMIX was purified by nickel affinity chromatography and ion exchange chromatography. The PfPMIX was flash frozen and stored at 80ºC until further use.

### Substrate peptide K_m_ determination

The K_m_ of Rh2N (DABCYL-HSFIQEGKEE-EDANS synthesized by Chinapeptides) was determined by titrating the fluorogenic peptide in 12-point 2-fold dilution series (0.08-80 µM) against each PMX enzyme at their optimal concentration (0.25 to 2.5 nM depending on enzyme activity). All reagents were prepared in buffer containing 25 mM sodium acetate (pH 5.5) and 0.005% Tween-20. The assay was conducted in 20 µl volume in a 384 well black plate (Corning #3820) with each PMX enzyme reaction initiated by the addition of 10 µl of PMX enzyme to plate containing 10 µl of titrated Rh2N. The enzyme kinetic assay was allowed to proceed at 37 °C for 30 minutes in the PHERAstarFSX plate reader (BMG Labtech). Samples were excited at 340 nm and fluorescence emission measured at 480 nm at one minute interval. Linear slope (0–15min) was used to determine the initial velocity (RFU/min) at each Rh2N concentration and plotted in Graphpad Prism to calculate the substrate Km based on Michaelis-Menten enzyme kinetics. Reported Km values were calculated on the Mean ± SD of three independent datasets.

### Plasmepsin X fluorogenic assay

Compound potency assay against each PMX enzyme was conducted in 15 µl reaction volume. Prior to that each PMX enzyme was titrated at 10 µM Rh2N in a time-course manner to determine the reaction linearity and the optimal protein concentration (in bracket below) for use in compound testing. Compounds (synthesized by Wuxi) were plated with 16-point 3-fold dilution series starting at 10 µM in triplicates in the 384 well black plates (Corning #3820) using an Echo acoustic dispenser (Labcyte) with all wells backfilled to 150 nl DMSO (1% final) and repeated in three independent experiments. Compounds were pre-incubated with 7.5 µl PMX enzyme (0.1 nM PMX, 0.1 nM PMXS315P, 0.1 nM PMXD245N, 0.2 nM PMXS359P, 0.05 nM PMXI363L, 0.025 nM PMXD245N/ PMXI363L) at room temperature for 20 mins and the reaction initiated with 7.5 µl Rh2N (10 µM final). Plate was incubated in a humidified incubator at 37 °C for 4 hours. Fluorescence output was measured in a PHERAstar Microplate Reader (BMG LABTECH) at excitation 340 nm and emission 480 nm. Data were normalized to percent inhibition relative to 1% DMSO (high control) and 1 µM WM382 (low controls) and plotted in Graphpad Prism. IC_50_ values were calculated using a nonlinear regression four-parameter fit analysis. The equation used is sigmoidal dose response (variable slope), Y = Bottom + (Top – Bottom) / (1 + (IC_50_ / X)^HillSlope^). Compound Ki values were calculated from the IC_50_ values using the Cheng-Prusoff equation, Ki = IC_50_/(1 + [S]/Km). Reported Ki values were calculated on the Mean ± SD of three independent dataset.

### PMX and PMIX primary screening method

PMX and PMIX primary screening were conducted in 1536 well plate (black low volume non-binding plate, Corning #3728) at a single compound dose of 10 nM in duplicates in 5 µl assay volume using in-house produced PfPMX (0.2 nM) or PfPMIX (5 nM) recombinant proteins. 1536 well assay ready plates were prepared using an Echo acoustic dispenser (Labcyte) with 5nl of 10 µM compound (1% DMSO) transferred into the 1536 well plate. 2.5 µl recombinant protein (PMX or PMIX) was dispensed into compound containing assay plates. Plates were allowed to incubate for 20 min at room temperature, and the reaction was started with a further 2.5 µl addition of 1.8 µM PMX substrate (i.e., Rh2N, DABCYL-HSFIQEGKEE-EDANS) or 7.2 µM PMIX substrate (i.e., RON3, DABCYL-KEISFLERRE-EDANS). Reaction was incubated at 37 °C for 2 hours. Enzyme and substrate working solutions were prepared in assay buffer consisting of 25 mM sodium acetate, 0.005% Tween-20, pH 5.5 for PMX assay and same buffer plus 100 mM sodium chloride for PMIX assay. Samples were excited at 340 nm and fluorescence emission measured at 480 nm using a PHERAstarFSX plate reader (BMG Labtech). Data were normalized to percent inhibition using positive and negative controls. The 0% inhibition control contained 1% DMSO and the 100% inhibition control contained 1 µM WM382. 931 hits were selected for activity confirmation in 5-points/10-fold dilution series in the same assays. IC_50_ values (relative inflection of dose response curve) were calculated by Dotmatics 5.3 and Spotfire 7.11.1 software using a nonlinear regression four-parameter fit analysis. The equation used is sigmoidal dose response (variable slope), Y = Bottom + (Top – Bottom) / (1 + (IC_50_ / X)^HillSlope^).

### *In vitro* selection of resistant parasites and reversion line

Three replicate cultures of clonal *P. falciparum* 3D7 parasites were grown on incremental increases of compound beginning at a concentration of 2x EC_50_. When parasitemia was significantly reduced, compound pressure was removed and when parasitemia recovered, compound pressure was resumed. This was repeated until the parasites were adapted to higher drug concentration over the course of up to 15 months. For the reversion line, drug pressure was removed from the resistant lines and cultures were maintained in drug-free complete media for 6 months. EC_50_ values were determined from these lines and genomic DNA was purified (QIAGEN Blood and Tissue Kit) for WGS analysis.

### Genome sequencing and bioinformatic analysis

An input of 100 ng of *P. falciparum* genomic DNA was prepared and indexed for Illumina sequencing using the TruSeq DNA sample Prep Kit (Illumina) as per manufacturer’s instruction. The library was quantified using the 4200 TapeStation System (Agilent). The indexed libraries were pooled and diluted to 750 pM for paired end sequencing (2x 150 bp reads) on a NextSeq 2000 instrument using the P2 300 cycle kit (Illumina) as per manufacturer’s instructions.

The quality of sequencing was confirmed using FastQC. Fastq files were aligned to reference PlasmoDB-52_Pfalciparum3D7 or PlasmoDB-57_PfalciparumDd2 as appropriate, using bwa-mem version v0.7.17. Sequences were filtered using Picard MarkDuplicates. Copy number analysis was performed using the R package QDNAseq v1.28.0 [45]. Structural variant calling was performed using GRIDSS v2.12.1 [46,47]. Regions of interest were inspected with Integrated Genome Viewer. Copy number bins in QDNASeq were filtered to exclude telomere and centromere regions, and by regions with mappability less than 50% using 30mers generated by GenMap [48]. Structural variant calls were used to determine the structure and genomic location of amplified regions. Calling of single nucleotide variants (SNVs) and indels was performed with bcftools v1.13 and calls were filtered using criteria: QUAL > 50; not an indel with length > 5 of A and T only; not in telomere, centromere or hypervariable regions [49]; not in genes described as PfEMP1, rifin, stevor or pseudogene. For each experiment in this study, we report all non-synonymous SNVs and indels in coding regions which were present in a majority of technical replicate samples, and not in parent lines.

A survey was done of 5,970 samples of *P. falciparum* from MalariaGEN *Plasmodium falciparum* Community Project (Pf6) [50], which contains curated genome variation data on *P. falciparum* samples from studies in 28 malaria-endemic countries. None of the 4 *plasmepsin X* SNVs reported in this study were present in the dataset (S315P, D245N, I363L, S359P).

Whole genome sequence data for this study have been deposited in the European Nucleotide Archive (ENA) at EMBL-EBI under accession numbers PRJEB36069 and PRJEB107760. Parental lines also used in previous studies are available under PRJEB53576 and PRJEB58099.

### Risk of resistance and minimum inoculum for resistance (MIR)

Cultures of 10^7^, 10^8^ and 10^9^ *P. falciparum* Dd2 parasites were exposed to ~1x EC_90_ (WM382: 1.5 nM, WM09: 1.5 nM, WM4: 80 nM, WM76: 2 nM, WM92: 1.5 nM, Atovaquone: 5nM, KAF156: 20 nM) of constant drug pressure and monitored for parasite recrudescence for 60 days. Experiments were carried out in triplicate and monitored by weekly microscopic examination of thin blood films. Media and compound were replaced three times each week. Evaluation of EC_50_ and genome analysis from WM92-treated recrudescing parasites was carried out as outlined above.

### Antimalarial resistome barcoding assay (AReBar)

A parasite pool consisting of 52 barcoded lines (see S1 Table) covering a range of mutant drug targets and resistance mechanisms was used to profile compounds for cross-resistance, essentially as described [19,26]. Briefly, triplicate cultures of the pool were exposed to 3 × EC_50_ of each compound for 14 days, with parasite growth measure by flow cytometry (stained with 1× SYBR Green and 200nM Mitotracker Deep Red). A drug-free control and treatment with the ATP4 inhibitor KAE609 were also included. The relative abundance of each line was measured by amplicon sequencing (Illumina platform) of the barcode region. Due to the absence of growth for cultures treated with WM382, WM4, WM92, WM76, WM09 or WM42, insufficient sample was obtained to generate an amplicon, in contrast to the drug-free and KAE609-treated parasite cultures.

### *P. falciparum* fitness cost assays

Synchronized ring-stage WM92_Resistant and WM92_Reversion parasite lines were mixed 1:1 with a 3D7 WT line. Synchronized ring-stage PMX(D245N), PMX(I363L) and PMX(D245N, I363L) lines were mixed 1:1 with a PMX(WT)-HA parental line. The fitness cost of the copy number increase of *pmx* and PMX SNVs were then evaluated as previously described by lactate dehydrogenase growth assays [19] with a starting titration series from 10 nM of WM92.

### Lead contact and materials availability

Further information and requests for resources and reagents should be directed to and will be fulfilled by the Lead Contact, Alan F. Cowman (cowman@wehi.edu.au). All unique/stable reagents generated in this study are available from the Lead Contact with a completed Materials Transfer Agreement.

## Supporting information

S1 Fig Confirmation of PMX(D245N) and PMX(I363L) in Pf3D7-WM92-Ramp up (RU) and Pf3D7-WM92-RU-Revertant (REV) parasites by PCR. The *pmx* gene was amplified by PCR (left) and sequenced (right), showing the presence of both mutations in Pf3D7-WM92-RU and Pf3D7-WM92-RU-REV parasites.
(TIF)

S2 Fig Long-term selection (LTS) of parasites with WM4 results in a copy number increase and a SNV in PMX. **A.** Wildtype parasites were exposed to increasing amounts of WM4 up to 250 nM over two years, generating the resistant line Pf3D7-WM4-Ramp up (RU)-LTS that had a resulting EC_50_ shift of 156-fold. **B-C.** Whole genome sequencing revealed a 14x copy number amplification in a region encompassing *pmx*. **C.** A single nucleotide variant was also found in PMX, resulting in PMX(S315P). A representative clone is shown in panel C. Error bars in panel A represent the standard deviation of two biological replicates, each with three technical replicates. Statistical analysis performed via an unpaired t-test with * indicating p < 0.05.
(TIF)

S3 Fig Generation of mutant parasite lines. **A.** CRISPR Cas9 cloning scheme to introduce the D245N, S315P, S359P, I363L and D245N,I363L mutations into *P. falciparum* 3D7 PMX. **B.** Confirmation western blot demonstrating the presence of Hemagglutinin (HA) tags on the amended parasite lines. **C.** 3’ Integration PCRs of the amended *pmx* gene. Sequenced PCR products showed single base-pair changes were present resulting in desired mutations in *pmx* coding sequence.
(TIF)

S4 Fig Mutations PMX(D245N) and PMX(D245N,I363L) but not PMX(I363L) alone results in parasite fitness cost. CRISPR engineered parasite lines (Pf3D7-CR-PMX) containing PMX mutations were mixed 50:50 with 3PMX(WT) parasites and EC_50_’s were evaluated by lactate dehydrogenase assays (LDH) on Day 0 and 28, alongside individual counterpart lines. Error bars indicate the SD of two technical replicates. Statistical analysis was performed in GraphPad Prism via a two-way ANOVA with multiple comparisons between day 0 and 28 time points. No asterisks indicate no significance. *** indicates p < 0.001, **** indicates p < 0.0001.
(TIF)

S1 Raw Gel Raw images of uncropped gels. S1 Fig. The pmx gene was amplified by PCR to identify the presence of mutations in Pf3D7-WM92-RU and Pf3D7-WM92-RU-REV parasites using the 3D7 sequence as the unmutated parental line as control. S3B Fig. Confirmation of PMX-HA, WT (parental), D245N, S315P, S359P, 136L and D245N+I363L *P. falciparum* parasites lines express a PMX protein that has a haemagglutinin tag. Also shown are the anti-HSP70 immunoblots as loading controls for the same parasite lines. S3C Fig. Uncropped agarose gel of PCR products to show 3’ integration of the amended *pmx* gene. Sequenced PCR products showed single base-pair changes were present resulting in desired mutations in *pmx* coding sequence.
(PDF)

S1 Table Parasite lines in starting pool of AReBar assay. (DOCX)

S2 Table Summary of parasite lines generated and utilized in this study. (DOCX)

S3 Table Summary of variants found by whole genome sequencing. (DOCX)

S4 Table Summary of data for parasite lines and recombinant proteins. (DOCX)

S5 Table Genes found in Chromosome 10 and 12 amplified regions in PfDd2-WM92-MIR parasites. (DOCX)
