# Supplementary material for: Dual plasmepsin IX and X inhibitors are refractory to development of resistance
Source: PLoS Pathog. 2026 Sep 8;22(9):e1014068. doi: 10.1371/journal.ppat.1014068 (PMC13568514; doi:10.1371/journal.ppat.1014068)
Supplement: Supplementary file 1 [file ppat.1014068.s005.pdf]

Uncropped gels for Supplementary Figures

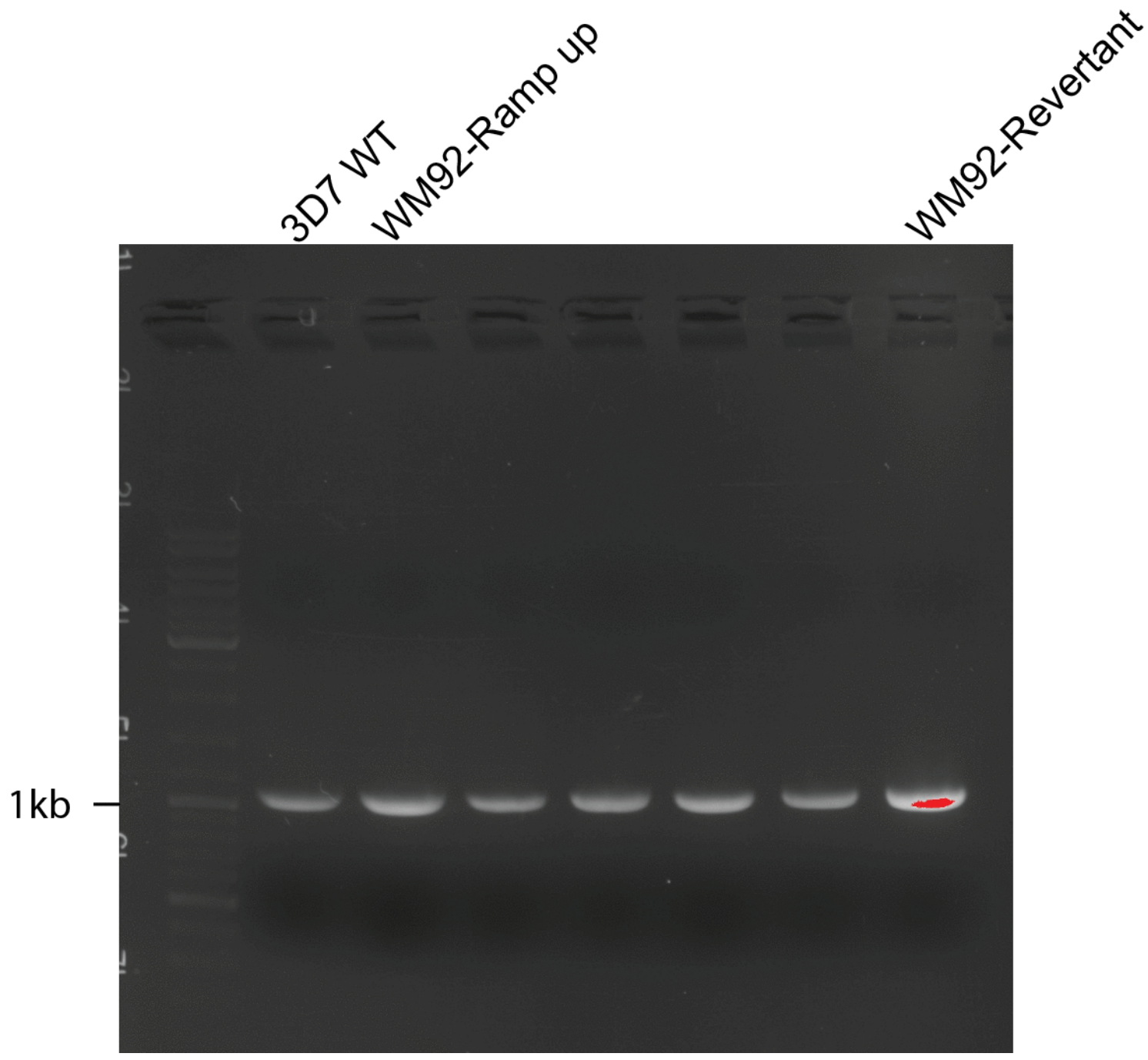

S1 Figure. Uncropped gel of the PCR results to confirm PMX(D245N) and PMX(I363L) in Pf3D7-WM92-Ramp up (RU) and Pf3D7-WM92-RU-Revertant (REV) parasites by PCR. The releveant tracks for 3D7wt, WM92-Ramp up and WM92-Revertant tracks are labelled. The *pmx* gene was amplified by PCR as above and sequenced (right panel in S1 Figure) that showed the presence of both mutations in Pf3D7-WM92-RU and Pf3D7-WM92-RU-REV parasites.

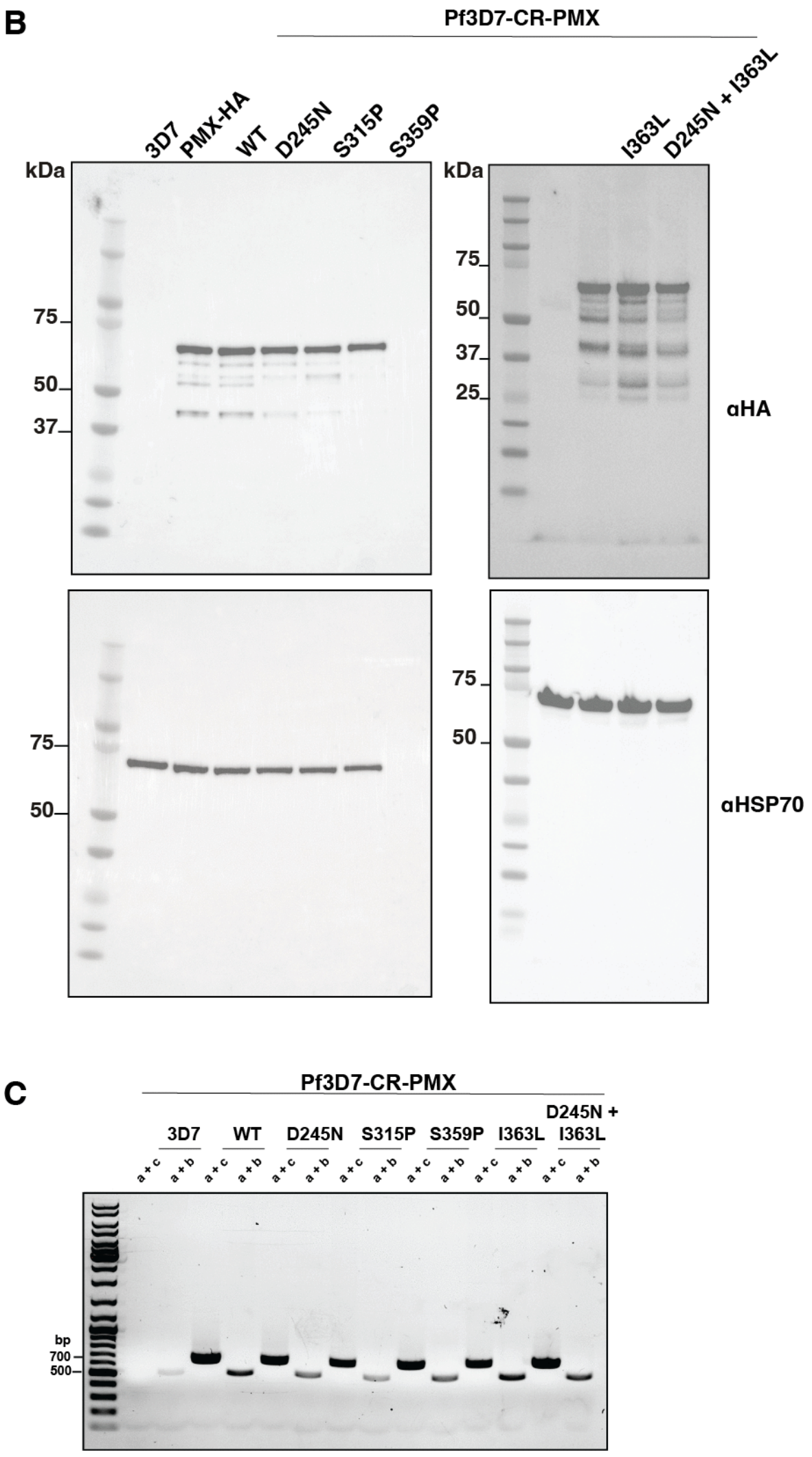

S3 Figure. Uncropped gels for generation of mutant parasite lines. B. Uncropped gels and immunoblots for confirmation to demonstrate the presence of Hemagglutinin (HA) tags on the amended parasite lines. **C.** Uncropped agarose gel to show 3' Integration of the amended *pmx* gene by PCR. Sequenced PCR products showed single base-pair changes were present resulting in desired mutations in *pmx* coding sequence.
