# Supplementary material for: Dual plasmepsin IX and X inhibitors are refractory to development of resistance
Source: PLoS Pathog. 2026 Sep 8;22(9):e1014068. doi: 10.1371/journal.ppat.1014068 (PMC13568514; doi:10.1371/journal.ppat.1014068)
Supplement: Supplementary file 2 [file ppat.1014068.s006.docx]

| Parasite line name | Input into starting pool (%) | PlasmoDB ID of resistance mechanism |
| --- | --- | --- |
| 3D7 | 15.35799111 | N/A |
| 3D7_ABCI3_R2180P | 9.328204191 | PF3D7_0319700 |
| 3D7_ACS10_M300I | 3.079176901 | PF3D7_0525100 |
| 3D7_ACS11_D648Y | 0.693246179 | PF3D7_1238800 |
| 3D7_ATPase2_CNV2 | 5.908663378 | PF3D7_1219600 |
| 3D7_DHFR-TS_G378E | 7.268427356 | PF3D7_0417200 |
| 3D7_DHFR-TS_I403L | 3.521040986 | PF3D7_0417200 |
| 3D7_FTb_A515T | 7.449774331 | PF3D7_1147500 |
| 3D7_MDR2_K840N | 3.324807319 | PF3D7_1447900 |
| 3D7_NCR1_A1108T | 7.804348267 | PF3D7_0107500 |
| Dd2 | 1.658174486 | N/A |
| Dd2_ATP4_G358S | 0.501410852 | PF3D7_1211900 |
| Dd2_ATP4_Q172H | 0.290290493 | PF3D7_1211900 |
| Dd2_AcAS_A597V | 0.19217366 | PF3D7_0627800 |
| Dd2_AcAS_T648M | 1.819898094 | PF3D7_0627800 |
| Dd2_AsnRS_R487S | 0.858014792 | PF3D7_0509600 |
| Dd2_CARL_I1139Kb | 0.070035119 | PF3D7_0321900 |
| Dd2_CARL_V1103L | 0.125860214 | PF3D7_0321900 |
| Dd2_CLK3_H259P | 1.283977183 | PF3D7_1114700 |
| Dd2_CPSF_Y408S_E | 0.534567575 | PF3D7_1438500 |
| Dd2_CPSF_Y408S_S | 0.216872035 | PF3D7_1438500 |
| Dd2_CRT_M343L | 0.667532801 | PF3D7_0709000 |
| Dd2_CSC1_L800P | 0.382993984 | PF3D7_1250200 |
| Dd2_DHFR-TS_S216R | 0.456750776 | PF3D7_0417200 |
| Dd2_DHODH_C276Y | 0.505132525 | PF3D7_0603300 |
| Dd2_DHODH_F227I | 0.115033529 | PF3D7_0603300 |
| Dd2_DHODH_I263F | 0.033833391 | PF3D7_0603300 |
| Dd2_DHODH_L531F | 0.215857034 | PF3D7_0603300 |
| Dd2_DNApolD_ATP4_L927V | 0.537950915 | PF3D7_1211900 |
| Dd2_GGPPS_S228T | 0.7253879 | PF3D7_1128400 |
| Dd2_HSP90_A41S | 1.194995365 | PF3D7_0708400 |
| Dd2_IleRS_E180D | 0.831624747 | PF3D7_1332900 |
| Dd2_IleRS_L810F | 0.552499273 | PF3D7_1332900 |
| Dd2_IleRS_V500A | 0.699336189 | PF3D7_1332900 |
| Dd2_MCP_D195N | 0.48652416 | PF3D7_0908800 |
| Dd2_MCP_P214T | 9.737249887 | PF3D7_0908800 |
| Dd2_PI4K_S1320L+L1418F | 0.930418248 | PF3D7_0509800 |
| Dd2_PI4K_S743F+H1484Y | 0.291643829 | PF3D7_0509800 |
| Dd2_PROTB5_A20V | 1.179093671 | PF3D7_1011400 |
| Dd2_PROTB5_M45I | 2.478634214 | PF3D7_1011400 |
| Dd2_ProRS_L482H | 0.024021708 | PF3D7_1213800 |
| Dd2_QRP1_D1863Y | 2.126766949 | PF3D7_1359900 |
| Dd2_TyrRS_S234C | 1.008235047 | PF3D7_1117500 |
| Dd2_UDP-GT_F37V | 0.101161839 | PF3D7_1113300 |
| Dd2_cytBC1_G33V | 0.332920566 | PF3D7_MIT02300 |
| Dd2_cytBC1_V284L | 0.325815554 | PF3D7_MIT02300 |
| Dd2_eEF2_L755F | 0.351528931 | PF3D7_1451100 |
| Dd2_eEF2_Y186N | 0.19995534 | PF3D7_1451100 |
| Dd2_kelch13_C580C | 0.874931487 | PF3D7_1343700 |
| Dd2_kelch13_C580Y | 0.946319942 | PF3D7_1343700 |
| Dd2_kelch13_R539T | 0.398895678 | PF3D7_1343700 |

**S1 Table. Parasite lines in starting pool of AReBar assay**
