# Supplementary material for: Dual plasmepsin IX and X inhibitors are refractory to development of resistance
Source: PLoS Pathog. 2026 Sep 8;22(9):e1014068. doi: 10.1371/journal.ppat.1014068 (PMC13568514; doi:10.1371/journal.ppat.1014068)
Supplement: Supplementary file 3 [file ppat.1014068.s007.docx]

**S2 Table. Resistant lines against PMX-selective inhibitors generated in this study.**

| **Parasite line** | **Selection compound** | **Selection method** | **EC_50_ (SD), nM** | **Fold-resistance from WT** | **Variant from WGS** |
| --- | --- | --- | --- | --- | --- |
| WM4_resistant | WM4 | Incremental increase | 66.6 (9.66) | 6X | 6x copy number in *pmx* |
| WM4_revertant | - | Off compound for  6 months | 41.7 (17.8) | 4X | 3x copy number in *pmx* |
| WM92_resistant | WM92 | Incremental increase | 34.8 (10.1) | 42X | PMX(D245N, I363L); 10x copy number in *pmx* |
| WM92_revertant | - | Off compound for  6 months | 30.7 (6.03) | 37X | PMX(D245N, I363L); 2x copy number in *pmx* |
| WM76_resistant | WM76 | Incremental increase | 23.5 (11.0) | 28X | 14x copy number in *pmx* |
| WM76_revertant | - | Off compound for  6 months | 9.67 (2.08) | 11X | 4x copy number in *pmx* |
| WM92-MIR | WM92 | Minimum Inoculum of Resistance | 8.87 (2.40) | 16X | DD2-PMX(S347P) |
| WM4_long-term selected | WM4 | Incremental increase | 653 (173) | 157X | PMX(S315P); 14x copy number in *pmx* |
