# Supplementary material for: Dual plasmepsin IX and X inhibitors are refractory to development of resistance
Source: PLoS Pathog. 2026 Sep 8;22(9):e1014068. doi: 10.1371/journal.ppat.1014068 (PMC13568514; doi:10.1371/journal.ppat.1014068)
Supplement: Supplementary file 4 [file ppat.1014068.s008.docx]

## **S3 Table. Summary of mutations found by whole genome sequencing.**

| **Experiment** | **Samples** | **Amplified region** | **Copy number** | **Missense Variants** |
| --- | --- | --- | --- | --- |
| **WM4_resistant** | WM4.1: 3 samples | Pf3D7_08:200..380 kb | 2 | PF3D7_0823400 V426E |
|  |  | Pf3D7_08:380..430 kb | 5 - 6 |  |
|  | WM4.2: 4 samples | Pf3D7_08:416..429 kb | 7 - 8 |  |
| **WM4_long-term selected** | WM4_LTS_S1 - WM4_LTS_S6: 6 samples | Pf3D7_08:160.. 398 kb | 2 | **PF3D7_0808200 (Plasmepsin X) S315P**  **AF 66-78%**  PF3D7_0823400 V426E  PF3D7_0935400 (GDV1) L45P |
|  |  | Pf3D7_08: 398..429 kb | 12-16 |  |
|  |  | Pf3D7_08:429..440 kb | 2 |  |
| **WM76_resistant** | WM76.2 | Pf3D7_08:409..425 kb | 18 |  |
|  | WM76.3 | Pf3D7_08:398..427 kb | 14 |  |
| **WM92_resistant** | WM92.02 and WM92.11 | Pf3D7_08:388..502 kb | 10 | **PF3D7_0808200 (Plasmepsin X) D245N^**  PF3D7_1026600 P377R |
| **WM92_MIR** | WM92_MIR_1.1, WM92_MIR_1.2, WM92_MIR_2.1, WM92_MIR_2.2 | PfDd2_10:260..291 kb | 2 | **PfDd2_080013400 (Plasmepsin X) S347P***  PfDd2_030013900 (SF3B1) A672T  PfDd2_130072000 I130V |
|  |  | PfDd2_12:1,533..1,551 | 2 |  |
| **WM4_revertant** | WM4_revert1 and WM4_revert2 | Pf3D7_08:160..399 kb | 1.3 | PF3D7_0823400 V426E |
|  |  | Pf3D7_08:399..429 kb | 3 - 3.5 |  |
|  |  | Pf3D7_08:429..440 kb | 1.3 |  |
| **WM76_revertant** | WM76_revert1 and WM76_revert2 | Pf3D7_08:398..427 kb | 4 |  |
| **WM92_revertant** | WM92_revert1 and WM92_revert2 | Pf3D7_08:409..429 kb | 2 | **PF3D7_0808200 (Plasmepsin X): D245N and I363L**  PF3D7_1026600 P377R |

All amplified regions with a copy number (CN) > 2 included the *plasmepsin x* gene, genomic location Pf3D7_08_v3:415,549..419,066(-) or PfDd2_08:413,183..414,868(-). This includes those where start position has been rounded to 416 kbp.

Missense variants are those present with high quality calls in all samples in group. Not all allele frequencies (AF) were calculated: where AF is not shown it was ≥ 99%, or Phred-scaled Likelihood (PL) was 256 for ALT and 0 for REF.

^An additional SNV, Plasmepsin X (I363L), was found in these samples via PCR.

*Equivalent to Plasmepsin X (S359P) in 3D7 parasites.
