# Supplementary material for: Dual plasmepsin IX and X inhibitors are refractory to development of resistance
Source: PLoS Pathog. 2026 Sep 8;22(9):e1014068. doi: 10.1371/journal.ppat.1014068 (PMC13568514; doi:10.1371/journal.ppat.1014068)
Supplement: Supplementary file 5 [file ppat.1014068.s009.docx]

**S4 Table. Summary of data for parasite lines and recombinant proteins**

|  | | | **EC_50_ (nM, AVE ± SD)** | | | | | | |
| --- | --- | --- | --- | --- | --- | --- | --- | --- | --- |
| **Parasite Line** | **Selected compound** | **Resistance MOA** | **Chloroquine** | **WM4^1^** | **WM92** | **WM76** | **WM382^1^** | **WM09** | **MK07602^2^** |
| Pf3D7 WT | - | - | 45.67±1.53 | 11.00±3.79 | 0.82±0.15 | 0.85±0.15 | 0.48±0.22 | 0.72±0.18 | 0.68±0.17 |
| Pf3D7-WM4-D2-RU^1^ | WM4 | *pmx* | 32.67±5.69 | 66.60±9.66 | 2.25±0.50 | 3.50±1.29 | 0.68±0.04 | 0.62±0.14 | 0.62±0.13 |
| Pf3D7-WM4-D2-RU-REV | - | *pmx* | nd | 41.67±17.79 | 1.63±1.19 | 3.00±1.00 | nd | nd | 0.76±0.22 |
| Pf3D7-WM76-RU-3 | WM76 | *pmx* | 30.00±10.58 | 415.00±262.0 | 72.25±11.96 | 23.50±11.03 | 0.91±0.03 | 0.74±0.29 | 0.82±0.13 |
| Pf3D7-WM76-RU-3-REV | - | *pmx* | nd | 240.00±108.2 | 59.33±18.58 | 9.67±2.08 | nd | nd | 0.93±0.08 |
| Pf3D7-WM92-RU | WM92 | *pmx* | 45.33±3.51 | 74.00±8.04 | 34.75±10.15 | 3.25±0.50 | 0.51±0.15 | 0.41±0.08 | 0.41±0.11 |
| Pf3D7-WM92-RU-3-REV | - | *pmx* | nd | 58.33±19.09 | 30.67±6.03 | 3.67±1.16 | nd | nd | 0.71±0.21 |
| PfDd2-WM92-MIR | WM92 | *pmx* | 453.30±25.17 | 10.67±1.53 | 16.33±4.04 | 0.73±0.12 | 0.43±0.22 | 0.36±0.12 | 0.46±0.05 |
| Pf3D7-MK-7602-RU^2^ | MK-7602 | *pmix/pmx* | nd | nd | nd | nd | nd | 3.67±1.16 | 9.25±1.50 |
| PH1263-C | Chloroquine | *pfcrt, pfmdr1, pfdhfr, pfdhps, pfK13, plasmepsin II* | 420.00±28.29 | 13.00±2.83 | 0.91±0.14 | 1.50±0.71 | 0.37±0.14 | 0.70±0.21 | 0.57±0.01 |
| Pf3D7-CR-PMX(WT) | - | PMX(WT) | 43.67±1.53 | 4.00±0.00 | 0.34±0.04 | 0.51±0.03 | 0.33±0.06 | 0.66±0.11 | 0.38±0.06 |
| Pf3D7-CR-PMX(S359P) | - | PMX(S359P) | 42.33±0.58 | 4.00±1.00 | 3.33±0.58 | 0.32±0.11 | 0.45±0.14 | 0.46±0.09 | 0.50±0.09 |
| Pf3D7-CR-PMX(D245N,I363L) | - | PMX(D245N,I363L) | 42.67±0.58 | 3.33±0.58 | 2.00±0.00 | 0.22±0.04 | 0.33±0.04 | 0.38±0.14 | 0.46±0.09 |
| Pf3D7-CR-PMX(D245N) | - | PMX(D245N) | 43.33±1.53 | 4.33±0.58 | 1.00±0.00 | 0.42±0.09 | 0.28±0.04 | 0.52±0.14 | 0.59±0.20 |
| Pf3D7-CR-PMX(I363L) | - | PMX(I363L) | 42.00±1.00 | 3.33±0.58 | 0.31±0.01 | 0.15±0.03 | 0.25±0.01 | 0.50±0.01 | 0.29±0.02 |
| Pf3D7-CR-PMX(S315P) | - | PMX(S315P) | 44.33 ±1.53 | 42.3±2.08 | 3.00±0.00 | 1.67±0.58 | 0.59±0.14 | 0.54±0.14 | 0.65±0.31 |
|  | | | | | | | | | |
|  | | | K_i_ (nM, AVE ± SD) | | | | | | |
| **Recombinant Protein** | **Origin** | **Platform** | **Chloroquine** | **WM4^1^** | **WM92** | **WM76** | **WM382^1^** | **WM09** | **MK-7602^2^** |
| rPfPMX(WT) | Newly generated | HEK 293 | nd | 0.70±0.20 | 0.02±0.01 | nd | nd | nd | nd |
| rPfPMX(S359P) | Newly generated | HEK 293 | nd | 5.15±1.82 | 6.94±1.14 | nd | nd | nd | nd |
| rPfPMX(D245N,I363L) | Newly generated | HEK 293 | nd | 4.20±1.04 | 0.18±0.05 | nd | nd | nd | nd |
| rPfPMX(D245N) | Newly generated | HEK 293 | nd | 4.06±1.44 | 0.98±0.25 | nd | nd | nd | nd |
| rPfPMX(I363L) | Newly generated | HEK 293 | nd | 7.12±0.48 | 9.39±0.69 | nd | nd | nd | nd |
| rPfPMX(S315P) | Newly generated | HEK 293 | nd | 30.27±2.99 | 2.87±0.38 | nd | nd | nd | nd |
| rPfPMX(WT) | Previously reported^1^ | sf21 insect cells | nd | 0.44±0.11 | 0.05±0.01 | 0.05±0.03 | 0.01±0.00 | 0.01±0.00 | nd |
| rPfPMIX(WT) | Previously reported^1^ | sf21 insect cells | nd | 306.00±16.00 | 75.00±12.90 | 16.20±7.36 | 0.65±0.20 | 0.15±0.06 | nd |

^1^ Favuzza et al. CHM (2020) PMID: 32109369

^2^ Favuzza et al. eBiomedicine (2026) PMID: 41353980

nd: not determined
