# Supplementary material for: Dual plasmepsin IX and X inhibitors are refractory to development of resistance
Source: PLoS Pathog. 2026 Sep 8;22(9):e1014068. doi: 10.1371/journal.ppat.1014068 (PMC13568514; doi:10.1371/journal.ppat.1014068)
Supplement: Supplementary file 6 [file ppat.1014068.s010.docx]

**S5 Table. Genes found in Chromosome 10 and 12 amplified regions in PfDd2-WM92-MIR parasites**

**Genes in the Chr 10 amplified regions**

| **Gene ID** | **Genomic Location (strand)** | **Product Description** |
| --- | --- | --- |
| PfDd2_100012400 | PfDd2_10: 259,213 - 260,909 (+) | conserved membrane protein, unknown function |
| PfDd2_100012500 | PfDd2_10: 261,109 - 262,027 (-) | conserved Plasmodium protein, unknown function |
| PfDd2_100012600 | PfDd2_10: 262,934 - 265,370 (-) | rhoGAP GTPase, putative |
| PfDd2_100012700 | PfDd2_10: 268,392 - 270,536 (+) | RING zinc finger protein, putative |
| PfDd2_100012800 | PfDd2_10: 271,462 - 272,121 (+) | conserved Plasmodium protein, unknown function |
| PfDd2_100012900 | PfDd2_10: 272,907 - 273,233 (+) | conserved Plasmodium protein, unknown function |
| PfDd2_100013000 | PfDd2_10: 273,821 - 274,150 (+) | conserved Plasmodium protein, unknown function |
| PfDd2_100013100 | PfDd2_10: 275,858 - 280,771 (-) | transcription factor with AP2 domain(s) (ApiAP2) |
| PfDd2_100013200 | PfDd2_10: 286,272 - 289,391 (-) | conserved Plasmodium protein, unknown function |

**Genes in the Chr 12 amplified regions**

| **Gene ID** | **Genomic Location (strand)** | **Product Description** |
| --- | --- | --- |
| PfDd2_120042200 | PfDd2_12:1542122-1543844(+) | conserved Plasmodium protein, unknown function |
| PfDd2_120042300 | PfDd2_12:1544779-1549818(-) | conserved Plasmodium protein, unknown function |
| PfDd2_120042000 | PfDd2_12:1533796-1539285(+) | conserved Plasmodium protein, unknown function |
| PfDd2_120042100 | PfDd2_12:1539509-1540821(-) | conserved Plasmodium protein, unknown function |
