# Supplementary material for: Dual plasmepsin IX and X inhibitors are refractory to development of resistance
Source: PLoS Pathog. 2026 Sep 8;22(9):e1014068. doi: 10.1371/journal.ppat.1014068 (PMC13568514; doi:10.1371/journal.ppat.1014068)
Supplement: Supplementary file 7 [file ppat.1014068.s012.docx]

**Reviewers' Comments:**

Reviewer's Responses to Questions

**Part I - Summary**
Please use this section to discuss strengths/weaknesses of study, novelty/significance, general execution and scholarship.

Reviewer #1: This is a comprehensive study that seeks to characterize resistance mechanisms for a series of antimalarial compounds that inhibit the proteases, PMX IX/X and which show potential for latter human trials(WM-7602, PMC12721278). Although the compound series and its antimalarial activity have been described in a number of other publications, here they provide a detailed evaluation of resistance mechanisms. They show they are unable to create resistant parasites for some dual protease inhibitors and they find novel single nucleotide variants in the PMX gene for some compounds. The authors transfer some of these SNVs into a clean genetic background and re-evaluate them in genetic isolation. In addition, the authors profile the compounds against panels of parasites with known mutations as well as parasites bearing mutations in PMX.

The strength of the work is that there is significant material, most of which appears to make sense and this is very comprehensive study, involving multiple members of the chemical series. The claim that some compounds are refractory to resistance development is supported by the data. The weaknesses is that it can be sometimes difficult to tell what is novel, given that new and previously published work from the same group is intermingled. For example, selections with WM4 were previously published (Cell Host Microbe. 2020 Apr 8;27(4):642–658.e12. doi: 10.1016/j.chom.2020.02.005) and these also showed CNVs in chromosome 8 that look similar to the ones shown here. Is the same data shown in this manuscript? The names of strains are often vague making it challenging to tell what is new and what is old. I note similar work was recently published on WM-7602 ([https://pmc.ncbi.nlm.nih.gov/articles/PMC12721278/](https://urldefense.com/v3/__https:/pmc.ncbi.nlm.nih.gov/articles/PMC12721278/__;!!KKt0acMt!eOWPJtOaRT5qLEJZkkkSE1dElq6B4XtdNHKuQVDh2vbA4vtKBiEcaffXL3PKbswsOO4MveUkbgsndWsDRw$))). The potential duplication includes phenotypic and biochemical assays with various compounds. Furthermore, the authors generally do not provide enough details or primary data in the tables and legends for the reviewer to recreate or follow their work. The authors mention this study will allow parasites clinical isolates to be screened for pre-existing resistance against PMX/IX inhibitors but do not explore non-PMX modulators of resistance (although this could be beyond the scope of the study). While the AREBar resistance panel is useful, it generally does not work for discovery of novel mechanisms.

**Major points.**

The authors should create an adequate strain/clone table with exact names of lines or clones that were phenotyped and/or sequenced and/or constructed (including the CRISPR lines). Once names are established, they should use these specific unique names in all text, figures and supplemental material (e.g. This stain table would include columns with the complete genotype, but also the genetic background (3D7, Dd2), the type of selection that was performed (e.g. six months ramp-up, MIR), the exact identity of compound that was used, and whether the line or clone was independently derived or was pulled from the same flask/well as another clone. It should indicate if cloning was performed. It should indicate the line/clone was genetically modified by CRISPR. Names should match names in the ENA sequence archive. It should also include a column indicating if the strain was made and characterized specifically for this study, or if it has been previously reported. If previously reported, a reference should be given. Right now, the reviewer is expected to read all the other papers and indirectly sort this out.

Authors response: We thank the reviewer for these helpful suggestions and have incorporated all recommendations to improve the clarity and consistency of parasite line nomenclature throughout the manuscript. To facilitate interpretation, we have created an updated supplementary table (Table S2) summarizing the relevant parasite line information, including making the distinction of the origin of the lines (newly generated or from previous studies) and have updated the nomenclature in the text and figures accordingly. Unfortunately, as sequences have already been uploaded to ENA, we cannot change the naming in the database. Instead, we have updated Table S3 to include the ‘new’ parasite line name and corresponding sample name for ENA with associated accession number to allow for ease of traceability of genome sequences.

I would also recommend a second excel file for biochemical and parasite phenotypes for clones or strains against different compounds. This should also distinguish new and old data, including for PMX biochemical assays. It could also be useful to have a compound Table giving references or indicating if the compound is new for this study or was previously described.

Authors response: We have adopted this suggestion by creating a new supplementary table (Table S4) that summarizes all phenotypic and biochemical data for the compounds evaluated against both parasite lines and recombinant proteins in this study. For compounds that have been reported previously, we have included references to the relevant publications. In addition, Figure 1 and associated legend has been updated to include citations to prior studies where appropriate, clearly distinguishing newly described compounds from those reported previously.

The author should also provide additional sequencing information instead of interpretted data. What genes are inside the the CNVs that are shown in Figure 3C, which are not mentioned in the text? These seem to be on chromosome 3, 10? What other SNVs were found in some of the lines? Were any mutations in major drug resistance alleles detected? If the goal is to understand the complete set of mutations that could give PMIX/X resistance, then the authors should list all the SNVs that were detected in any clone at 100% allele coverage, not just the ones that appeared in PMX or which appeared in multiple selections, given that the latter tend to derive from founder effects. With this much whole genome sequencing data, it is expected that there will be other independent modulators of drug resistance. This will increase the long term impact of the study.

Authors response: We thank the reviewer for pointing out this discrepancy between Figure 3 and the genome variants discussed in text. An error was identified in the CNV figure, where a version scaled to the Dd2 parental line was inadvertently used, creating the appearance of a CNV event on chromosome 3. These figures have been corrected with the unscaled versions which removed this event in chromosome 3. However, as the reviewer pointed out, they also contain amplification events at chromosome 10 and 12. These genes amplified in these regions can now be found in Table S5. The results text has been altered as below:

Line 389: *“Unlike the resistance mechanism observed in the incremental selection experiments (performed with 3D7 P. falciparum parasites), no copy number variation of the pmx gene was detected in the Dd2 WM92-selected line (Fig 3C). However, a small ~2 copy number was detected in chromosomes 10 and 12 of the PfDd2-WM92-MIR parasites (Fig 3C, S3_Table), with 13 unrelated genes amplified in these regions (S5 Table).*

The amplified events in chromosome 5 and 7 are not of interest as they were also found to be present in the Dd2 parental line. This has also been referred to in the text on Line 400: *“Amplification events identified on chromosomes 5 and 7 were excluded from further analysis because they were already present in the parental Dd2 strain.”*

Additional SNVs identified in the genomes can be found in Table S3 and this has also been made clearer in the results on Line 402: “Additionally, three SNVs were identified, one in *pmx* that resulted in an S347P amino acid change, which is equivalent to the S359P position in 3D7 parasites (S3 Table).”

Could the authors also comment on whether any of the observed evolved PMX SNVs have been observed in sequenced field isolates like those characterized in the MalariaGen projects?

Authors response: We thank the reviewer for this valuable suggestion. We had in fact performed this analysis during the study but did not clearly report the findings in the original manuscript. We have now included the methodological details of this analysis in the Materials and Methods section [Lines 935-942] and added a discussion of these findings [Lines 697-703], highlighting that the PMX resistance-associated variants identified under experimental drug selection do not appear to be circulating in natural parasite populations at detectable frequencies. These observations support the conclusion that such mutations are likely to require specific drug-selective pressure to emerge.

Discussion at lines 697-703 now reads: “*Importantly, interrogation of 5,970 P. falciparum genomes from the MalariaGEN Plasmodium falciparum Community Project, encompassing isolates from 28 malaria-endemic countries, revealed that none of the PMX resistance-associated variants identified in this study (S315P, D245N, I363L, and S359P) were present in the dataset. This finding indicates that these mutations are not currently circulating at detectable frequencies in natural parasite populations and supports the notion that PMX resistance requires specific selective pressure to emerge.*”.

Material and Methods at Lines 943-947 now reads: “*A survey was done of 5,970 samples of P. falciparum from MalariaGEN Plasmodium falciparum Community Project, which contains curated genome variation data on Plasmodium falciparum samples from studies in 28 malaria-endemic countries. None of the 4 plasmepsin X SNVs reported in this study were present in the dataset (S315P, D245N, I363L, S359P).*”.

**Minor points.**

Please rotate compounds in Figure 1 so SAR is evident. Please provide parasite EC50s under the compound. Please also show structure of clinical candidate, WM-7602, given that it is mentioned.

Authors response: Figure 1 now has been updated so all compounds are in the same orientation, EC_50_ values are directly below structures and MK-7602 structure has been included.

I was confused because Table S1 says that WM92 resistant parasites contain “PMX(D245N, I363L);10x copy number in pmx” but then Figure 3 shows no CNV in PMX and a SNP, S347P. The EC50s also don’t seem to match? Better nomenclature should help.
Authors response: The two variants highlighted by the reviewer arose from independent resistance selection experiments using the same compound (WM92). One variant was generated through a ramp-up (RU) selection approach, resulting in the PMX(D245N, I363L) mutations and a 10-fold amplification of *pmx*, whereas the second was generated using the minimum inoculum for resistance (MIR) method, resulting in the PMX(S347P) mutation. To avoid confusion, we have revised the nomenclature throughout the manuscript and now refer to these lines as *Pf3D7-WM92-RU* and *PfDd2-WM92-MIR*, respectively.

The figure legends are placed inside the text but the figures are at the end, which is a strange type of arrangement, and which makes reviewing difficult because it is hard to find the figure legends.

Authors response: The placement of figure legends within the manuscript text and the figures at the end of the document follows the formatting requirements of PLOS Pathogens for manuscript submission. We appreciate that this arrangement can make reviewing less convenient and thank the reviewer for their patience. We are, of course, happy to follow any alternative formatting requested by the journal during subsequent stages of the review and production process.

Reviewing would also be much easier if there were better labels on panels in Figures. For Figure 3C, for example, why not write WM92 resistant clone 1 and WM-92 resistant clone 2. Or if better names are established, use these. For example, names like the following might be used: Pf3D7-WM2-F1-C1 or PfDd2-WM92-MIR-1.2 in figures and tables. Sample names seem a bit better in their ENA deposited files but don’t match names in supplemental files.

Authors response: This figure now reflects the new naming nomenclature- PfDd2-WM92-MIR-1 and PfDd2-WM92-MIR-2 which will assist on readability. Unfortunately, we are unable to change the naming of ENA deposited files but have updated Table S3 with names used within the manuscript and names of sequences uploaded to ENA to assist with traceability.

I don’t understand the difference between Table S2 and S3.

Authors response: An updated Table S2 now contains more information about parasite lines generated and utilised in this study and the updated Table S3 now contains a greater detailing of the genetic variants present in these lines.

It is also not clear what type of WGS SNV filtering that was done. The authors mention that they only retained SNVs that were found in all samples, which is a very strict filtering. You would not expect the same important SNV in all “samples” especially if the samples were independently derived? Or were samples just technical repeats of sequencing? The authors state that the objective is to understand the different mechanisms of resistance, if they only focus on PMX, they are missing out on understanding the roles of transporters or possibly other secondary targets. Strains or clones that were sequenced are not well described in the text.

Authors response: We thank the reviewer for this important comment and agree that additional detail regarding the whole-genome sequencing analysis and filtering strategy improves the interpretation of the genetic data.

To address this point, we have expanded the Materials and Methods section [Lines 937-942] to provide a detailed description of the variant-calling and filtering pipeline. Specifically, SNVs and indels were called using bcftools v1.13 and filtered according to the following criteria: QUAL > 50; exclusion of low-complexity indels consisting of >5 A/T bases; exclusion of variants located within telomeric, centromeric, hypervariable regions, and genes annotated as var (PfEMP1), rifin, stevor, or pseudogenes. Structural variant calls were additionally used to define the structure and genomic location of amplified regions.

We would also like to clarify that the filtering criterion was not intended to require the same SNV to be present across independently derived resistant lines. Rather, for each individual selection experiment, variants were retained if they were present in the majority of technical replicate sequencing samples and absent from the corresponding parental line.

We agree with the reviewer that the identification of additional adaptive mutations beyond *pmx* gene itself could provide valuable insights into resistance mechanisms, including potential compensatory pathways, transporter-mediated effects, or secondary targets. For this reason, our analysis was not restricted to *pmx* but we assessed all non-synonymous coding-region SNVs and indels that passed the filtering criteria described above, and all variants identified in the resistant lines are reported in Table S3.

Across the independently derived resistant lines, however, the only recurrent genetic changes consistently associated with the resistance phenotype were *pmx* amplification and/or *pmx* coding mutations. No recurrent mutations were identified in transporter genes or other *loci* that would support an alternative resistance mechanism. Together with the genetic validation studies presented in the manuscript, these findings support *pmx* as the primary determinant of the observed resistance phenotypes.

Methods: It is not clear from the methods if SNVs were identified based on subtraction of SNVs from isogenic parent sequences? In this case, it could be hard to find SNVs except for those in candidate genes, such as PMX. If there were thousands of candiate SNVs, were other genes over-represented with missense mutations?

Authors response: As outlined in our response above, we have expanded the whole-genome sequencing methods [Lines 921-942] to provide additional detail regarding the variant-calling and filtering strategy.

Specifically, candidate SNVs were identified relative to the corresponding isogenic parental line and were required to be present in the majority of technical replicate sequencing samples while being absent from the parent. Additional quality and genomic-location filters were then applied, as detailed in the revised Materials and Methods section.

We would also like to clarify that our analysis was not restricted to *pmx* or other candidate genes. Rather, all non-synonymous coding-region SNVs and indels passing the filtering criteria were assessed, and all identified variants are reported in Table S3. Although additional mutations were detected, we did not observe recurrent enrichment of missense mutations in any genes other than *pmx* across independent selections. In contrast, *pmx* amplification and/or *pmx* coding mutations represented the only consistent genetic changes associated with the resistance phenotype, supporting their central role in mediating resistance.

Some European Nucleotide Archive Sequences that were deposited seem to be associated with the older publication on dual targeting and the identities of other samples don’t seem to match identities that are given in this manuscript. The reference PRJEB53576 cites “P. falciparum cipargamin resistance with PfATP4 mutations?

Authors response: This was an error and accession numbers for this study should only be PRJEB36069 and PRJEB107760. This has been corrected in the methods section (Lines 948-951) and Table S3 has also been updated to include this information.

Columns in Table S1 need more explanation.

Authors response: Column titles in Table S1 have now been updated to read ‘Parasite line name’, ‘Input into starting pool (%)’ and ‘PlasmoDB ID of resistance mechanism’.

Reviewer #2: This is an excellent article by Favuzza et al. I have only minor comments:

The authors refer to artemisinin resistance. As per the WHO, the term should consistently be artemisinin partial resistance.

Authors response: We thank the reviewer for this important comment. We agree that the terminology should be consistent with current WHO recommendations. Accordingly, we have revised the manuscript throughout and replaced the term "artemisinin resistance" with "artemisinin partial resistance" where appropriate [Lines 65, 69, 69/70, 668].

Introduction: The authors state “Both WM382 and MK-7602 target the liver, blood and transmission stages of P. falciparum and they have a high barrier for the selection of resistance due to their dual targeting of PMIX and PMX [17, 19].” It would help readers to know the mean (or range of) IC50 values against these different stages, and specify when these compounds are active (e.g. early vs mature gametocytes, or liver stages, specifying which asexual blood stages).

Authors response: We directly referenced the manuscripts (17 and 19) showing this data and have now inserted a range from these publications as requested by the reviewer as below

Line 109-111: Both WM382 and MK-7602 target the liver (<2 x 100 mg/kg blocks *P. berghei* *in vivo*), blood (~0.4 nM inhibits asexual stage development *in vitro*) and transmission stages of *P. falciparum* (2-10 nM blocks oocyst formation) and they have a high barrier for the selection of resistance due to their dual targeting of PMIX and PMX [17, 19].

One line 109 the authors refer to promising CHMI data. Can they be more specific? Is this work under review or in press?

Authors response: This work has undergone peer review and is currently in revisions, as such “under review” has been added to Line 117.

One line 116 the authors refer to PfCARL as a resistance determinant for ganaplacide. They should also mention that PfACT and PgUGT have also been identified.

Authors response: We thank the reviewer for realising this omission. PfACT and PfUGT have now been added as resistance mechanisms for Ganaplacide. Line 123 now reads: *“In P. falciparum this approach has successfully identified mutations in PfCARL, PfACT and PfUGT as resistance mechanism(s) for the imidazolopiperazine compound Ganaplacide, and mutations in PfATP4 conferring resistance to several new antimalarial candidates including Cipargamin* [24, 25]*”.*

Table 1: the requisite detail is often in the text. One addition would be to stipulate in the table that the EC50 fold pressure was “maximum EC50 fold pressure”.

Authors response: This column title now reads *“Maximum EC_50_ fold pressure’’* in Table 1 on Line 279.

When describing instances when parasites amplified the pmx locus and also acquired a SNP, it would help to list estimates of what percentage of amplified loci carried a SNP. For example with the WM382-presured resistant line that acquired ~10 copies of a segment including pmx, approximately how many carried a SNP. This is even more relevant when discussing the revertants that deamplified the locus but retained SNPs.

Authors response: We thank the reviewer for highlighting this important point. This information is currently included in Table S3, whereby Allele Frequency (AF) of SNVs are that <99% are reported in the last column with the footnote specifying *“Not all allele frequencies (AF) were calculated: where AF is not shown it was ≥99% or Phred-scaled Likelihood (PL) was 256 for ALT and 0 for REF”.*

For example, in the Pf3D7-WM4-RU-LTS parasite line, the PMX(S315P) SNV has an AF of 66-78%, meaning its present in ~70% of extra copies of the gene.

We realize that this information was not made clear in results section and, as such, have made the following additions to specify SNV frequency in lines:

Line 319: *“Whole genome sequencing also demonstrated both D245N and I363L were present in both copies of pmx in the Pf3D7-WM92-RU-REV parasites (S3_Table).”*

*Line 338: “Whole genome sequencing of this long-term selected line revealed a 14x copy number increase surrounding the pmx gene, with an additional SNV identified as S315P in PMX that was found in ~70% of extra copies of pmx (S2B, C Fig, S3_Table).”*

Figure 3 needs clarification. The legend states that various inocula were tested in triplicate. Yet for Atovaquone and WM4 for example, Figure 3A shows (n=4) and (n=2). Please clarify. Also the MIR calculation needs work. If only some of the inocula yielded resistance then a value would never be 10^7 or 10^8. Also, the way to describe MIR is different here (is a parasite inoculum) than table 1 that shows Log10 MIR values.

Authors response: Each inoculum (10^7^, 10^8^ and 10^9^) was tested in three independent selection dishes (or ‘triplicate’. The ‘n=x’ that was present in the table in Figure 3A referred to the number of experiments that had been performed for that compound in the MIR assay including historical data (i.e. Atovaquone has been tested across n=4 separate biological experiments, each in triplicate for each inoculum). To avoid confusion, we have now removed the ‘n=x’ from the table and kept the original statement in the figure legend on Line 371: *“The initial inoculum of P. falciparum parasites was varied from 10^7^, 10^8^ or 10^9^ in triplicate”.* We have also adjusted the MIR values in Figure 3A to match Table 1, presenting Log10 MIR values.

The fitness data are unusual in that they present the change in EC50 but not a percent change in growth rate per asexual blood stage generation. Can the authors extrapolate mean fitness costs per 48 hr ABS generation?

Authors response: We thank the reviewer for this suggestion. These experiments were primarily designed to assess whether increased *pmx* copy number imposes a fitness cost on parasite growth, with the impact of PMX point mutations being a secondary consideration. As copy number-associated fitness effects may require multiple replication cycles to become apparent, we elected to use a long-term competition assay in which mutant and wild-type parasites were mixed 1:1 and cultured for 28 days, with population shifts inferred from EC_50_ measurements at Day 0 and Day 28.

Importantly, no significant difference was observed at Day 0 between the mixed cultures and mutant parasites alone (EC_50_ >10 nM; Fig. 6A), indicating that the assay was not biased by the initial parasite composition. While the 72-hour LDH assay inherently spans a single intraerythrocytic cycle and therefore provides some assessment of short-term growth, our experimental design was not intended to sensitively detect subtle fitness differences within a single 48-hour cycle. Additional approaches, such as allele-specific qPCR or direct competition assays with more frequent sampling, would be required to definitively assess fitness costs over a single replication cycle.

To improve clarity for readers, we have revised the discussion to explicitly state the duration of the competition growth assay. Line 732 now reads: *“Competition assays demonstrated that resistant parasites were significantly outcompeted by wild-type parasites in the absence of drug pressure over 28 days.”*

Can the authors mention whether pmix amplification has never been observed by any group. Can the authors speculate why pmix cannot amplify yet pmx can.

Authors response: An amplification in *pmix* has been previously observed with resistant parasites to the clinical candidate MK-7602 (Favuzza et al. 2026). To obtain resistant parasites, this took 15 months of continuous selection which resulted in 20 copies of *pmix* and 18 copies of *pmx.* In this manuscript this information has been cited in Table S4 and in the discussion on Line 598: *“*previously described MK-7602 selected parasite line (‘Pf3D7-MK-7602-RU’) with amplified copies of both *pmx* and *pmix* [19]*”.*

However, we acknowledge that this was not sufficiently clear in the original Results section and have now provided greater emphasis on this point on Line 239: *“In stark contrast, dual PMIX/X inhibitors WM09 and WM42 demonstrated a resistance profile similar to WM382 rather than the clinical candidate MK-7602, as neither decreased sensitivity nor resistance could be generated even after prolonged selection (Table 1) [17].* *Although resistance to MK-7602 has previously been generated, this required an exceptionally high level of gene amplification (20 copies of pfpmix and 18 copies of pfpmx) and approximately 15 months of continuous selection [19], highlighting the substantial resistance barrier associated with dual PMIX/X inhibition.”*

Table S2 now also includes this MK-7602 resistant line ‘Pf3D7-MK-7602-RU’ and the associated genotype of the increase copies in both *pmix* and *pmx.*

Interestingly, we never have identified a PMIX-selective inhibitor and therefore have never observed resistance mechanisms associated with PMIX alone – either amplifications or SNVs. We believe this is due to our discussion point on Line 740: *“The inability to design PMIX-selective inhibitors during the medicinal chemistry program was noteworthy and likely reflects fundamental differences in the structural architecture of the S' binding sites between PMIX and PMX. The large loop structure in PfPMX, and its associated conformational mobility, likely creates a more permissive S' pocket that tolerates substantial structural variation. Conversely, dual PMIX/X inhibitors achieve their broad efficacy by concentrating their interactions within the S pockets - regions that are typically more critical for substrate binding and thus more structurally conserved across both proteases.”*

In the Discussion, the authors mention that resistance to Ganaplacide, Cipargamin or DSM265 has been obtained within 3-4 months of continuous drug pressure. They used a ramping approach (the starting pressure should be listed). The authors should also cite work showing that resistance to each compound was also obtained using single step selection procedures that last one month.

Authors response: We thank the reviewer for this helpful comment and for highlighting that our original statement was overly general and did not adequately reflect the different selection methodologies used to generate resistance to these compounds. We agree that resistance to Ganaplacide, Cipargamin, and DSM265 has been obtained using distinct experimental approaches, including both single-step selection procedures and prolonged exposure to increasing drug concentrations.

To improve accuracy and provide a more balanced summary of the available literature, we have revised the text in the Discussion and now reads Lines 643–646: "*For several clinical-stage candidates, including Ganaplacide, Cipargamin, and DSM265, resistant parasites have been selected within 1–4 months of drug pressure, either through single-step selection at high drug concentrations or following prolonged exposure to incrementally increasing sub-lethal concentrations.*"

We believe this revised wording more accurately captures the range of experimental approaches used to select resistant parasites and addresses the reviewer's concern while maintaining a concise summary within the Discussion.

May labs use only 0.5% Albumax, as opposed to the 5% described herein. Are the authors sure they used 5%?

Authors response: We thank the reviewer for carefully identifying this error and bringing it to our attention. We confirm that the concentration of Albumax used in these experiments was 0.5%, not 5%. The value reported in the manuscript was a typographical error.

The corresponding Methods section has been corrected and now reads: "*0.5% Albumax II™ (Gibco) + 5% heat-inactivated human serum or 1% Albumax II™ (Gibco)*" [Lines 767–768].

Reviewer #3: This study investigated the resistance potential of plasmepsin X (PMX)-selective inhibitors and dual plasmepsin IX/X (PMIX/X) inhibitors in Plasmodium falciparum, aiming to evaluate their promise as new antimalarial candidates in the context of emerging resistance to artemisinin-based combination therapies (ACTs). The authors characterized a panel of PMX-selective and dual PMIX/X inhibitors regarding parasite growth inhibition and enzymatic activity. Through long-term in vitro selection experiments, the study demonstrated that these compounds possess a high barrier to resistance, although parasites with reduced sensitivity to PMX-selective inhibitors could still be generated.
This is a high-quality study with very well-designed and carefully executed experiments. The work provides important advances for the malaria field, particularly in the development of novel antimalarial inhibitors with improved resistance profiles. Furthermore, the detailed investigation of the molecular mechanisms underlying resistance emergence is highly relevant and significantly contributes to our understanding of parasite adaptation and strategies to overcome antimalarial drug resistance.

**Part II – Major Issues: Key Experiments Required for Acceptance**
Please use this section to detail the key new experiments or modifications of existing experiments that should be absolutely required to validate study conclusions.

Generally, there should be no more than 3 such required experiments or major modifications for a "Major Revision" recommendation. If more than 3 experiments are necessary to validate the study conclusions, then you are encouraged to recommend "Reject".

Reviewer #1: I don't have that many key experiments that the reviewers need to do. However, the inadequate presentation makes this hard to review for novelty.

Reviewer #2: No additional experiments required

Reviewer #3: It is not clear to me what the novel contribution of Figure 1 is, or whether it represents a compilation of previously published data. The authors could clarify this point more explicitly in the text.
In the discussion, I felt that the manuscript lacks a deeper consideration of the relationship between compound structure–activity and the barrier to resistance generation. In particular, WM92 presented the lowest MIR value among the compounds evaluated, while also representing one of the most potent inhibitors in the study. A discussion addressing how specific structural features may influence both potency and susceptibility to resistance selection would considerably strengthen the manuscript.

Authors response: We acknowledge that, in the present study, forward genetic analyses were conducted on only a limited subset of compounds, which constrains our ability to comprehensively define the structural features that contribute to resistance mitigation. Expanding these analyses to a broader panel of analogues or systematically screening diverse compounds against PMX-resistant mutants, would likely provide deeper insights into the relationship between compound structure and resistance or sensitivity profiles. Additionally, given the success of approaches such as deep mutational scanning in elucidating resistance mechanisms in other proteases (e.g., SARS-CoV-2 PLpro and Mpro), we consider this a promising future direction for PMX. Such studies could enable a more detailed understanding of how specific mutations impact compound binding and resistance evolution.

As such we have made this addition to the discussion (Line 717-722): *“While resistance selection and forward genetic analyses were performed on only a subset of compounds in this study, expanding these investigations to a broader panel of PMX-selective analogues would provide greater insight into the relationship between compound structure and resistance propensity. Future approaches, including deep mutational scanning of PMX, may further define the structural determinants that influence compound susceptibility and resistance evolution.”*

For this class of compounds, very little has been explored regarding their activity against circulating Plasmodium vivax and Plasmodium falciparum isolates worldwide, and understanding the activity profile of these compounds in this context is crucial and currently represents a limitation. In the study (DOI: 10.1016/j.ebiom.2025.106061), only a limited number of P. vivax isolates were evaluated, and I could not find information regarding assays performed with P. falciparum field isolates. Such experiments are fundamental for understanding the activity of these compounds against circulating resistant isolates and for better assessing their translational potential in endemic settings.

**Part III – Minor Issues: Editorial and Data Presentation Modifications**
Please use this section for editorial suggestions as well as relatively minor modifications of existing data that would enhance clarity.

Reviewer #1: As described above, the authors need to be clearer about how strains were derived for this study and use better nomenclature if they would like others to be able to reproduce their work.

Authors response: To improve clarity and reproducibility, we have revised the nomenclature of all parasite lines throughout the manuscript to more clearly reflect their derivation and selection method. These updated names have been applied consistently throughout the text and figures. In addition, we have updated supplementary tables (Tables S2 and S4) that now provide a clear summary of parasite line origins, nomenclature, and associated phenotypic and biochemical data. We believe these changes substantially improve the traceability of parasite line derivation and will facilitate interpretation and reproduction of the work by other researchers.

Reviewer #2: Minor:
Line 345: Should be “line” not “lines”

Authors response: We thank the reviewer for identifying this typographical error. The text has been corrected, and "lines" has been changed to "line" [Line 352].

Line 715: “datasets” not “dataset”.

Authors response: The text has been corrected, and "dataset" has been changed to "datasets" [Line 869].

Reviewer #3: I suggest that the authors include and discuss the following important reference in the field DOI: 10.1016/j.pt.2021.04.006.

Authors response: We thank the reviewer for highlighting this important reference. In fact, our *in vitro* resistance studies were designed and interpreted in accordance with the framework proposed by Duffey and colleagues (2021). We agree that this was not explicitly stated in the original manuscript and we believe that explicitly referencing this framework better contextualises the favourable resistance profile of plasmepsins inhibitors.

To address this point, we have now included a paragraph in the Discussion section [Lines 651–666] that places our findings within the context of this resistance-risk assessment framework.

The added text reads: *“Consistent with this observation, we assessed the resistance liability of these compounds using the in vitro candidate selection risk assessment framework, which has been proposed as a standardised approach for prioritising next-generation antimalarials. Based on the resistance-selection data generated in this study, all dual PMIX/X inhibitors and the majority of PMX-selective inhibitors would be categorised as low-risk compounds. WM92 represented the sole exception and would be classified as a medium-risk compound. These findings compare favourably with several clinically deployed antimalarials and highlight the high barrier to resistance associated with PMIX/X inhibition. Collectively, our results further strengthen the rationale for developing dual PMIX/X inhibitors as durable components of future combination regimens aimed at delaying or preventing the emergence of clinical resistance.”.*
