# Supplementary material for: Dual plasmepsin IX and X inhibitors are refractory to development of resistance
Source: PLoS Pathog. 2026 Sep 8;22(9):e1014068. doi: 10.1371/journal.ppat.1014068 (PMC13568514; doi:10.1371/journal.ppat.1014068)
Supplement: Peer review history

# Author comment: Author response to Decision Letter 0

**DOI:** 10.1371/journal.ppat.1014068.r001

**Transfer Alert**

This paper was transferred from another journal. As a result, its full editorial history (including decision letters, peer reviews and author responses) may not be present.

8 Mar 2026

# Peer review: Decision Letter 0

**DOI:** 10.1371/journal.ppat.1014068.r002

**Authors:** Tracey J. Lamb, Maria Belen Cassera

26 May 2026

PPATHOGENS-D-26-00611

Dual plasmepsin IX and X inhibitors are refractory to development of resistance

PLOS Pathogens

Dear Dr. Cowman,

Thank you for submitting your manuscript to PLOS Pathogens. After careful consideration, we feel that it has merit but does not fully meet PLOS Pathogens's publication criteria as it currently stands. Therefore, we invite you to submit a revised version of the manuscript that addresses the points raised during the review process.

Please submit your revised manuscript by July 31. If you will need more time than this to complete your revisions, please reply to this message or contact the journal office at plospathogens@plos.org. When you're ready to submit your revision, log on to https://www.editorialmanager.com/ppathogens/ and select the 'Submissions Needing Revision' folder to locate your manuscript file.

Please include the following items when submitting your revised manuscript:

* A letter that responds to each point raised by the editor and reviewer(s). You should upload this letter as a separate file labeled 'Response to Reviewers'. This file does not need to include responses to any formatting updates and technical items listed in the 'Journal Requirements' section below.

* A marked-up copy of your manuscript that highlights changes made to the original version. You should upload this as a separate file labeled 'Revised Manuscript with Track Changes'.

* An unmarked version of your revised paper without tracked changes. You should upload this as a separate file labeled 'Manuscript'.

If you would like to make changes to your financial disclosure, competing interests statement, or data availability statement, please make these updates within the submission form at the time of resubmission. Guidelines for resubmitting your figure files are available below the reviewer comments at the end of this letter.As the corresponding author, your ORCID iD is verified in the submission system and will appear in the published article. PLOS supports the use of ORCID, and we encourage all coauthors to register for an ORCID iD and use it as well. Please encourage your coauthors to verify their ORCID iD within the submission system before final acceptance, as unverified ORCID iDs will not appear in the published article. Only the individual author can complete the verification step; PLOS staff cannot verify ORCID iDs on behalf of authors.

We look forward to receiving your revised manuscript.

Kind regards,

Maria Belen Cassera, PhD

Guest Editor

PLOS Pathogens

Tracey Lamb

Section Editor

PLOS PathogensSumita Bhaduri-McIntosh

Editor-in-Chief

PLOS Pathogens

orcid.org/0000-0003-2946-9497Michael Malim

Editor-in-Chief

PLOS Pathogens

orcid.org/0000-0002-7699-2064

**Additional Editor Comments**

The reviewers find the manuscript valuable, comprehensive, and potentially impactful, with strong experimental work and important implications for antimalarial development. However, they also identify substantial issues in presentation and clarity, particularly regarding the distinction between new and previously published data (make clearer the novelty of this work), strain/clone nomenclature, supporting data transparency, and figure/table organization. These concerns are addressable through revision and improved documentation without new experimental work.

**Journal Requirements:**

If the reviewer comments include a recommendation to cite specific previously published works, please review and evaluate these publications to determine whether they are relevant and should be cited. There is no requirement to cite these works unless the editor has indicated otherwise.

1) We ask that a manuscript source file is provided at Revision. Please upload your manuscript file as a .doc, .docx, .rtf or .tex. If you are providing a .tex file, please upload it under the item type u2018LaTeX Source Fileu2019 and leave your .pdf version as the item type u2018Manuscriptu2019.

2) Please provide an Author Summary. This should appear in your manuscript between the Abstract (if applicable) and the Introduction, and should be 150-200 words long. The aim should be to make your findings accessible to a wide audience that includes both scientists and non-scientists. Sample summaries can be found on our website under Submission Guidelines:

https://journals.plos.org/plospathogens/s/submission-guidelines#loc-parts-of-a-submission

3) We do not publish any copyright or trademark symbols that usually accompany proprietary names, eg ©,  ®, or TM  (e.g. next to drug or reagent names). Therefore please remove all instances of trademark/copyright symbols throughout the text, including:

- TM on pages: Page no 25, line number 614, and 615.

4) Please upload all main figures as separate Figure files in .tif or .eps format. For more information about how to convert and format your figure files please see our guidelines:

https://journals.plos.org/plospathogens/s/figures

5) Please upload a copy of Figures 1-6 which you refer to in your text on pages 6, 10, 13, 15, 17, and 18. Or, if the figure is no longer to be included as part of the submission please remove all reference to it within the text.

6) In the online submission form, you indicated that "The data that support the findings of this study are available from the corresponding author upon reasonable request". All PLOS journals now require all data underlying the findings described in their manuscript to be freely available to other researchers, either

1. In a public repository

2. Within the manuscript itself

3. Uploaded as supplementary information.

This policy applies to all data except where public deposition would breach compliance with the protocol approved by your research ethics board. If your data cannot be made publicly available for ethical or legal reasons (e.g., public availability would compromise patient privacy), please explain your reasons by return email and your exemption request will be escalated to the editor for approval. Your exemption request will be handled independently and will not hold up the peer review process, but will need to be resolved should your manuscript be accepted for publication. One of the Editorial team will then be in touch if there are any issues.

7) Please amend your detailed Financial Disclosure statement. This is published with the article. It must therefore be completed in full sentences and contain the exact wording you wish to be published.

1) Please clarify all sources of financial support for your study. List the grants, grant numbers, and organizations that funded your study, including funding received from your institution. Please note that suppliers of material support, including research materials, should be recognized in the Acknowledgements section rather than in the Financial Disclosure

2) State the initials, alongside each funding source, of each author to receive each grant. For example: "This work was supported by the National Institutes of Health (####### to AM; ###### to CJ) and the National Science Foundation (###### to AM)."

3) State what role the funders took in the study. If the funders had no role in your study, please state: "The funders had no role in study design, data collection and analysis, decision to publish, or preparation of the manuscript."

4) If any authors received a salary from any of your funders, please state which authors and which funders..

If you did not receive any funding for this study, please simply state: u201cThe authors received no specific funding for this work.u201d

8. Please send a completed 'Competing Interests' statement, including any COIs declared by your co-authors. If you have no competing interests to declare, please state "The authors have declared that no competing interests exist". Otherwise please declare all competing interests beginning with the statement "I have read the journal's policy and the authors of this manuscript have the following competing interests:"

9. PLOS now requires that authors provide the original uncropped and unadjusted images underlying all blot or gel results reported in a submission’s figures or Supporting Information files. This policy and the journal’s other requirements for blot/gel reporting and figure preparation are described in detail at https://journals.plos.org/plosone/s/figures#loc-blot-and-gel-reporting-requirements and https://journals.plos.org/plosone/s/figures#loc-preparing-figures-from-image-files.

When you submit your revised manuscript, please ensure that your figures adhere fully to these guidelines and provide the original underlying images for all blot or gel data reported in your submission. See the following link for instructions on providing the original image data: https://journals.plos.org/plosone/s/figures#loc-original-images-for-blots-and-gels.

In your cover letter, please note whether your blot/gel image data are in Supporting Information or posted at a public data repository, provide the repository URL if relevant, and provide specific details as to which raw blot/gel images, if any, are not available. Email us at customercare@plos.org if you have any questions."

**Reviewers' Comments:**

Reviewer's Responses to Questions

**Part I - Summary**

Please use this section to discuss strengths/weaknesses of study, novelty/significance, general execution and scholarship.

Reviewer #1: This is a comprehensive study that seeks to characterize resistance mechanisms for a series of antimalarial compounds that inhibit the proteases, PMX IX/X and which show potential for latter human trials(WM-7602, PMC12721278). Although the compound series and its antimalarial activity have been described in a number of other publications, here they provide a detailed evaluation of resistance mechanisms. They show they are unable to create resistant parasites for some dual protease inhibitors and they find novel single nucleotide variants in the PMX gene for some compounds. The authors transfer some of these SNVs into a clean genetic background and re-evaluate them in genetic isolation. In addition, the authors profile the compounds against panels of parasites with known mutations as well as parasites bearing mutations in PMX.

The strength of the work is that there is significant material, most of which appears to make sense and this is very comprehensive study, involving multiple members of the chemical series. The claim that some compounds are refractory to resistance development is supported by the data. The weaknesses is that it can be sometimes difficult to tell what is novel, given that new and previously published work from the same group is intermingled. For example, selections with WM4 were previously published (Cell Host Microbe. 2020 Apr 8;27(4):642–658.e12. doi: 10.1016/j.chom.2020.02.005) and these also showed CNVs in chromosome 8 that look similar to the ones shown here. Is the same data shown in this manuscript? The names of strains are often vague making it challenging to tell what is new and what is old. I note similar work was recently published on WM-7602 (https://pmc.ncbi.nlm.nih.gov/articles/PMC12721278/)). The potential duplication includes phenotypic and biochemical assays with various compounds. Furthermore, the authors generally do not provide enough details or primary data in the tables and legends for the reviewer to recreate or follow their work. The authors mention this study will allow parasites clinical isolates to be screened for pre-existing resistance against PMX/IX inhibitors but do not explore non-PMX modulators of resistance (although this could be beyond the scope of the study). While the AREBar resistance panel is useful, it generally does not work for discovery of novel mechanisms.

Major points.

The authors should create an adequate strain/clone table with exact names of lines or clones that were phenotyped and/or sequenced and/or constructed (including the CRISPR lines). Once names are established, they should use these specific unique names in all text, figures and supplemental material (e.g. This stain table would include columns with the complete genotype, but also the genetic background (3D7, Dd2), the type of selection that was performed (e.g. six months ramp-up, MIR), the exact identity of compound that was used, and whether the line or clone was independently derived or was pulled from the same flask/well as another clone. It should indicate if cloning was performed. It should indicate the line/clone was genetically modified by CRISPR. Names should match names in the ENA sequence archive. It should also include a column indicating if the strain was made and characterized specifically for this study, or if it has been previously reported. If previously reported, a reference should be given. Right now, the reviewer is expected to read all the other papers and indirectly sort this out.

I would also recommend a second excel file for biochemical and parasite phenotypes for clones or strains against different compounds. This should also distinguish new and old data, including for PMX biochemical assays. It could also be useful to have a compound Table giving references or indicating if the compound is new for this study or was previously described.

The author should also provide additional sequencing information instead of interpretted data. What genes are inside the the CNVs that are shown in Figure 3C, which are not mentioned in the text? These seem to be on chromosome 3, 10? What other SNVs were found in some of the lines? Were any mutations in major drug resistance alleles detected? If the goal is to understand the complete set of mutations that could give PMIX/X resistance, then the authors should list all the SNVs that were detected in any clone at 100% allele coverage, not just the ones that appeared in PMX or which appeared in multiple selections, given that the latter tend to derive from founder effects. With this much whole genome sequencing data, it is expected that there will be other independent modulators of drug resistance. This will increase the long term impact of the study.

Could the authors also comment on whether any of the observed evolved PMX SNVs have been observed in sequenced field isolates like those characterized in the MalariaGen projects?

Minor points.

Please rotate compounds in Figure 1 so SAR is evident. Please provide parasite EC50s under the compound. Please also show structure of clinical candidate, WM-7602, given that it is mentioned.

I was confused because Table S1 says that WM92 resistant parasites contain “PMX(D245N, I363L);10x copy number in pmx” but then Figure 3 shows no CNV in PMX and a SNP, S347P. The EC50s also don’t seem to match? Better nomenclature should help.

The figure legends are placed inside the text but the figures are at the end, which is a strange type of arrangement, and which makes reviewing difficult because it is hard to find the figure legends.

Reviewing would also be much easier if there were better labels on panels in Figures. For Figure 3C, for example, why not write WM92 resistant clone 1 and WM-92 resistant clone 2. Or if better names are established, use these. For example, names like the following might be used: Pf3D7-WM2-F1-C1 or PfDd2-WM92-MIR-1.2 in figures and tables. Sample names seem a bit better in their ENA deposited files but don’t match names in supplemental files.

I don’t understand the difference between Table S2 and S3.

It is also not clear what type of WGS SNV filtering that was done. The authors mention that they only retained SNVs that were found in all samples, which is a very strict filtering. You would not expect the same important SNV in all “samples” especially if the samples were independently derived? Or were samples just technical repeats of sequencing? The authors state that the objective is to understand the different mechanisms of resistance, if they only focus on PMX, they are missing out on understanding the roles of transporters or possibly other secondary targets. Strains or clones that were sequenced are not well described in the text.

Methods: It is not clear from the methods if SNVs were identified based on subtraction of SNVs from isogenic parent sequences? In this case, it could be hard to find SNVs except for those in candidate genes, such as PMX. If there were thousands of candiate SNVs, were other genes over-represented with missense mutations?

Some European Nucleotide Archive Sequences that were deposited seem to be associated with the older publication on dual targeting and the identities of other samples don’t seem to match identities that are given in this manuscript. The reference PRJEB53576 cites “P. falciparum cipargamin resistance with PfATP4 mutations?

Columns in Table S1 need more explanation.

Reviewer #2: This is an excellent article by Favuzza et al. I have only minor comments:

The authors refer to artemisinin resistance. As per the WHO, the term should consistently be artemisinin partial resistance.

Introduction: The authors state “Both WM382 and MK-7602 target the liver, blood and transmission stages of P. falciparum and they have a high barrier for the selection of resistance due to their dual targeting of PMIX and PMX [17, 19].” It would help readers to know the mean (or range of) IC50 values against these different stages, and specify when these compounds are active (e.g. early vs mature gametocytes, or liver stages, specifying which asexual blood stages).

One line 109 the authors refer to promising CHMI data. Can they be more specific? Is this work under review or in press?

One line 116 the authors refer to PfCARL as a resistance determinant for ganaplacide. They should also mention that PfACT and PgUGT have also been identified.

Table 1: the requisite detail is often in the text. One addition would be to stipulate in the table that the EC50 fold pressure was “maximum EC50 fold pressure”.

When describing instances when parasites amplified the pmx locus and also acquired a SNP, it would help to list estimates of what percentage of amplified loci carried a SNP. For example with the WM382-presured resistant line that acquired ~10 copies of a segment including pmx, approximately how many carried a SNP. This is even more relevant when discussing the revertants that deamplified the locus but retained SNPs.

Figure 3 needs clarification. The legend states that various inocula were tested in triplicate. Yet for Atovaquone and WM4 for example, Figure 3A shows (n=4) and (n=2). Please clarify. Also the MIR calculation needs work. If only some of the inocula yielded resistance then a value would never be 10^7 or 10^8. Also, the way to describe MIR is different here (is a parasite inoculum) than table 1 that shows Log10 MIR values.

The fitness data are unusual in that they present the change in EC50 but not a percent change in growth rate per asexual blood stage generation. Can the authors extrapolate mean fitness costs per 48 hr ABS generation?

Can the authors mention whether pmix amplification has never been observed by any group. Can the authors speculate why pmix cannot amplify yet pmx can.

In the Discussion, the authors mention that resistance to Ganaplacide, Cipargamin or DSM265 has been obtained within 3-4 months of continuous drug pressure. They used a ramping approach (the starting pressure should be listed). The authors should also cite work showing that resistance to each compound was also obtained using single step selection procedures that last one month.

May labs use only 0.5% Albumax, as opposed to the 5% described herein. Are the authors sure they used 5%?

Reviewer #3: This study investigated the resistance potential of plasmepsin X (PMX)-selective inhibitors and dual plasmepsin IX/X (PMIX/X) inhibitors in Plasmodium falciparum, aiming to evaluate their promise as new antimalarial candidates in the context of emerging resistance to artemisinin-based combination therapies (ACTs). The authors characterized a panel of PMX-selective and dual PMIX/X inhibitors regarding parasite growth inhibition and enzymatic activity. Through long-term in vitro selection experiments, the study demonstrated that these compounds possess a high barrier to resistance, although parasites with reduced sensitivity to PMX-selective inhibitors could still be generated.

This is a high-quality study with very well-designed and carefully executed experiments. The work provides important advances for the malaria field, particularly in the development of novel antimalarial inhibitors with improved resistance profiles. Furthermore, the detailed investigation of the molecular mechanisms underlying resistance emergence is highly relevant and significantly contributes to our understanding of parasite adaptation and strategies to overcome antimalarial drug resistance.

**********

**Part II – Major Issues: Key Experiments Required for Acceptance**

Please use this section to detail the key new experiments or modifications of existing experiments that should be absolutely required to validate study conclusions.

Generally, there should be no more than 3 such required experiments or major modifications for a "Major Revision" recommendation. If more than 3 experiments are necessary to validate the study conclusions, then you are encouraged to recommend "Reject".

Reviewer #1: I don't have that many key experiments that the reviewers need to do. However, the inadequate presentation makes this hard to review for novelty.

Reviewer #2: No additional experiments required

Reviewer #3: It is not clear to me what the novel contribution of Figure 1 is, or whether it represents a compilation of previously published data. The authors could clarify this point more explicitly in the text.

In the discussion, I felt that the manuscript lacks a deeper consideration of the relationship between compound structure–activity and the barrier to resistance generation. In particular, WM92 presented the lowest MIR value among the compounds evaluated, while also representing one of the most potent inhibitors in the study. A discussion addressing how specific structural features may influence both potency and susceptibility to resistance selection would considerably strengthen the manuscript.

For this class of compounds, very little has been explored regarding their activity against circulating Plasmodium vivax and Plasmodium falciparum isolates worldwide, and understanding the activity profile of these compounds in this context is crucial and currently represents a limitation. In the study (DOI: 10.1016/j.ebiom.2025.106061), only a limited number of P. vivax isolates were evaluated, and I could not find information regarding assays performed with P. falciparum field isolates. Such experiments are fundamental for understanding the activity of these compounds against circulating resistant isolates and for better assessing their translational potential in endemic settings.

**********

**Part III – Minor Issues: Editorial and Data Presentation Modifications**

Please use this section for editorial suggestions as well as relatively minor modifications of existing data that would enhance clarity.

Reviewer #1: As described above, the authors need to be clearer about how strains were derived for this study and use better nomenclature if they would like others to be able to reproduce their work.

Reviewer #2: Minor:

Line 345: Should be “line” not “lines”

Line 715: “datasets” not “dataset”.

Reviewer #3: I suggest that the authors include and discuss the following important reference in the field DOI: 10.1016/j.pt.2021.04.006.

**********

PLOS authors have the option to publish the peer review history of their article (what does this mean?). If published, this will include your full peer review and any attached files.

If you choose “no”, your identity will remain anonymous but your review may still be made public.

**Do you want your identity to be public for this peer review?**  For information about this choice, including consent withdrawal, please see our Privacy Policy.

Reviewer #1: No

Reviewer #2: No

Reviewer #3: No

[NOTE: If reviewer comments were submitted as an attachment file, they will be attached to this email and accessible via the submission site. Please log into your account, locate the manuscript record, and check for the action link "View Attachments". If this link does not appear, there are no attachment files.]

**Figure resubmission:**

While revising your submission, we strongly recommend that you use PLOS’s NAAS tool (https://ngplosjournals.pagemajik.ai/artanalysis) to test your figure files. NAAS can convert your figure files to the TIFF file type and meet basic requirements (such as print size, resolution), or provide you with a report on issues that do not meet our requirements and that NAAS cannot fix.

After uploading your figures to PLOS’s NAAS tool - https://ngplosjournals.pagemajik.ai/artanalysis, NAAS will process the files provided and display the results in the "Uploaded Files" section of the page as the processing is complete. If the uploaded figures meet our requirements (or NAAS is able to fix the files to meet our requirements), the figure will be marked as "fixed" above. If NAAS is unable to fix the files, a red "failed" label will appear above. When NAAS has confirmed that the figure files meet our requirements, please download the file via the download option, and include these NAAS processed figure files when submitting your revised manuscript.

**Reproducibility:**

To enhance the reproducibility of your results, we recommend that authors of applicable studies deposit laboratory protocols in protocols.io, where a protocol can be assigned its own identifier (DOI) such that it can be cited independently in the future. Additionally, PLOS ONE offers an option to publish peer-reviewed clinical study protocols. Read more information on sharing protocols at https://plos.org/protocols?utm_medium=editorial-email&utm_source=authorletters&utm_campaign=protocols

# Author comment: Author response to Decision Letter 1

**DOI:** 10.1371/journal.ppat.1014068.r003

24 Jul 2026

Attachment Submitted filename: Authors response to reviewers.docx

# Peer review: Decision Letter 1

**DOI:** 10.1371/journal.ppat.1014068.r004

**Authors:** Tracey J. Lamb, Maria Belen Cassera

10 Aug 2026

Dear Dr. Cowman,

We are pleased to inform you that your manuscript 'Dual plasmepsin IX and X inhibitors are refractory to development of resistance' has been provisionally accepted for publication in PLOS Pathogens.

Before your manuscript can be formally accepted you will need to complete some formatting changes, which you will receive in a follow up email. A member of our team will be in touch with a set of requests.

Please note that your manuscript will not be scheduled for publication until you have made the required changes, so a swift response is appreciated.

IMPORTANT: The editorial review process is now complete. PLOS will only permit corrections to spelling, formatting or significant scientific errors from this point onwards. Requests for major changes, or any which affect the scientific understanding of your work, will cause delays to the publication date of your manuscript.

Should you, your institution's press office or the journal office choose to press release your paper, you will automatically be opted out of early publication. We ask that you notify us now if you or your institution is planning to press release the article. All press must be co-ordinated with PLOS.

Thank you again for supporting Open Access publishing; we are looking forward to publishing your work in PLOS Pathogens.

Best regards,

Maria Belen Cassera, PhD

Guest Editor

PLOS Pathogens

Tracey Lamb

Section Editor

PLOS Pathogens

Sumita Bhaduri-McIntosh

Editor-in-Chief

PLOS Pathogens

orcid.org/0000-0003-2946-9497

Michael Malim

Editor-in-Chief

PLOS Pathogens

orcid.org/0000-0002-7699-2064

***********************************************************

This is an elegant, well-supported work on a topic that will be greatly benefit the malaria field. The authors have done an excellent job addressing the reviewers’ comments, and the remaining reviewer suggestions are minor points that I hope the authors will consider as they prepare the final version of the manuscript.

It has been a pleasure to serve as Guest Editor for this important and thoughtfully prepared contribution to the field of antimalarials.

Reviewer Comments (if any, and for reference):

Reviewer's Responses to Questions

**Part I - Summary**

Please use this section to discuss strengths/weaknesses of study, novelty/significance, general execution and scholarship.

Reviewer #1: The authors present an exhaustive study of resistance mechanisms for a new class of Plasmepsin IX/X inhibitors. Overall, their conclusions are well supported by the provided data and the authors have been responsive to reviewer's concerns.

Reviewer #2: Earlier provided

**********

**Part II – Major Issues: Key Experiments Required for Acceptance**

Please use this section to detail the key new experiments or modifications of existing experiments that should be absolutely required to validate study conclusions.

Generally, there should be no more than 3 such required experiments or major modifications for a "Major Revision" recommendation. If more than 3 experiments are necessary to validate the study conclusions, then you are encouraged to recommend "Reject".

Reviewer #1: (No Response)

Reviewer #2: Earlier provided.

**********

**Part III – Minor Issues: Editorial and Data Presentation Modifications**

Please use this section for editorial suggestions as well as relatively minor modifications of existing data that would enhance clarity.

Reviewer #1: The legend to Table S3 is a bit ambiguous: Many samples with high plasmepsin x amplification also had a duplication (CN=2) on one or both flanks, which is not shown.

Dose response data from P. falciparum strain PH1263-C is listed in Table S4 but is not listed in Table S2 (strains used or created in this study). Usually a strain table has all strains/clones and a description of where they came from. It may also include strains from other species, including human cell lines used for toxicity testing or E. coli lines used for protein expression. In this respect, S1 and S2 could probably be combined.

Reviewer #2: This revised manuscript by Favuzza et al provides an excellent rebuttal to the earlier set of reviews. I was reviewer 2 and the authors have responded well to my earlier queries.

Two additional points:

• Lines 697-703 now read: “Importantly, interrogation of 5,970 P. falciparum genomes from the MalariaGEN Plasmodium falciparum Community Project, encompassing isolates from 28 malaria-endemic countries.

What version of the MalariaGEN data was used? The authors should report their analysis with Pf8, the most recent genome dataset.

• Also, in my earlier review, I wrote: “The fitness data are unusual in that they present the change in EC50 but not a percent change in growth rate per asexual blood stage generation. Can the authors extrapolate mean fitness costs per 48 hr ABS generation?”

The authors interpreted this as my asking what the fitness cost was in a single generation. The authors note that their competition assays lasted 28 days. I apologize for the confusion. I was asking if they could infer the percent fitness cost per generation that would result over a 14-generation assay in the change in prevalence of the mutant line from start to end. This can be calculated using the formula:

P’ = P((1-x)^n)

where P’ is equal to the proportion of the mutant line at the assay endpoint, P is equal to the proportion of that line on day 0, n is equal to the number of generations from the assay start to finish, and x is equal to the fitness cost per generation. For example, if a mutant line was at 50% on day 0, and 10% on day 28, and n = 14 generations, then the formula is

0.1 = 0.5 * ((1-x)^14). In this case x= 0.11, i.e. a 11% fitness cost per generation would reduce the starting proportion by ~80% from day 0 to day 28 (i.e from 50% to 10%), compared to the isogenic wild-type control.

I leave it to the authors’ discretion if they wish to estimate this value or leave the language as is.

**********

PLOS authors have the option to publish the peer review history of their article (what does this mean?). If published, this will include your full peer review and any attached files.

If you choose “no”, your identity will remain anonymous but your review may still be made public.

**Do you want your identity to be public for this peer review?**  For information about this choice, including consent withdrawal, please see our Privacy Policy.

Reviewer #1: No

Reviewer #2: No

# Editor report: Acceptance letter

**DOI:** 10.1371/journal.ppat.1014068.r005

**Authors:** Tracey J. Lamb, Maria Belen Cassera

Dear Prof. Cowman,

We are delighted to inform you that your manuscript, "Dual plasmepsin IX and X inhibitors are refractory to development of resistance," has been formally accepted for publication in PLOS Pathogens.

We have now passed your article onto the PLOS Production Department who will complete the rest of the pre-publication process. All authors will receive a confirmation email upon publication.

Thank you again for supporting open-access publishing; we are looking forward to publishing your work in PLOS Pathogens.

Best regards,

Dominique Soldati-Favre

Editor-in-Chief

PLOS Pathogens
